# An ADH toolbox for raspberry ketone production from natural resources via a biocatalytic cascade

**DOI:** 10.1007/s00253-021-11332-9

**Published:** 2021-05-14

**Authors:** Aileen Becker, Dominique Böttcher, Werner Katzer, Karsten Siems, Lutz Müller-Kuhrt, Uwe T. Bornscheuer

**Affiliations:** 1 Department of Biotechnology and Enzyme Catalysis, Institute of Biochemistry, University of Greifswald, Greifswald, Germany; 2 AnalytiCon Discovery GmbH, Potsdam, Germany

**Keywords:** Raspberry ketone, Biocatalysis, Enzymatic cascade, Natural products, Alcohol dehydrogenase toolbox

## Abstract

**Abstract:**

Raspberry ketone is a widely used flavor compound in food and cosmetic industry. Several processes for its biocatalytic production have already been described, but either with the use of genetically modified organisms (GMOs) or incomplete conversion of the variety of precursors that are available in nature. Such natural precursors are rhododendrol glycosides with different proportions of (*R*)- and (*S*)-rhododendrol depending on the origin. After hydrolysis of these rhododendrol glycosides, the formed rhododendrol enantiomers have to be oxidized to obtain the final product raspberry ketone. To be able to achieve a high conversion with different starting material, we assembled an alcohol dehydrogenase toolbox that can be accessed depending on the optical purity of the intermediate rhododendrol. This is demonstrated by converting racemic rhododendrol using a combination of (*R*)- and (*S*)-selective alcohol dehydrogenases together with a universal cofactor recycling system. Furthermore, we conducted a biocatalytic cascade reaction starting from naturally derived rhododendrol glycosides by the use of a glucosidase and an alcohol dehydrogenase to produce raspberry ketone in high yield.

**Key points:**

• *LB-ADH, LK-ADH and LS-ADH oxidize (R)-rhododendrol*

• *RR-ADH and ADH1E oxidize (S)-rhododendrol*

• *Raspberry ketone production via glucosidase and alcohol dehydrogenases from a toolbox*

**Graphical abstract:**

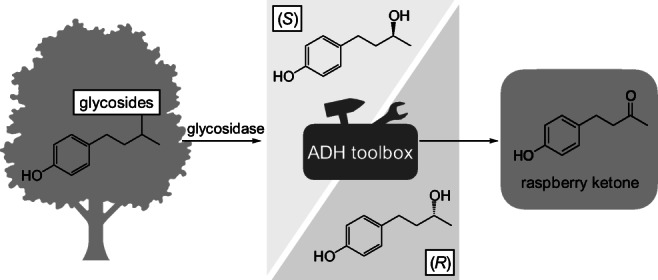

**Supplementary Information:**

The online version contains supplementary material available at 10.1007/s00253-021-11332-9.

## Introduction

Raspberry ketone (4-(4-hydroxyphenyl)-butan-2-one) is of high economical relevance (Wang et al. 2019) due to its characteristic scent and low odor threshold (Larsen and Poll 1990). The aroma compound is widely applied as flavoring agent in food industry for products like sweets, yoghurts, or soft drinks (Deifel 1989; Beekwilder et al. 2007; Wang et al. 2019; Milke et al. 2020). In addition, raspberry ketone is utilized as an attractant in insect baits (Perez 1983; Metcalf et al. 1983; Deifel 1989) and as a component in perfumes (Dumont et al. 1996; Farwick et al. 2019), whereas further applications in cosmetic industry, e.g., as skin whitening or hair growth inducing agent, remain controversial (Harada et al. 2008; Kim et al. 2016). Other publications promote dose-dependent health benefits with regard to an anti-obese effect (Morimoto et al. 2005; Park 2015; Tsai et al. 2017; Zhao et al. 2019; Mir et al. 2019) or suggest medical applications due to its anti-oxidant and anti-inflammatory potential (Parmar and Tripathi 1991; Khan et al. 2018; Fouad et al. 2019; Mohamed et al. 2020; Hamdy et al. 2020).

So far, the chemical-synthetic production of raspberry ketone is dominating the market. However, increasing consumer awareness is demanding for a naturally derived product, especially in food and cosmetic industry (Milke et al. 2020; Malik and Rawat 2021). Natural production methods do not only include the direct isolation from natural sources, but also the enzymatic or microbial bioconversion of natural precursors, according to EU regulations (Kosjek et al. 2003; European Parliament 2008; Schloesser and Lambert 2018; Milke et al. 2020).

As its natural occurrence in raspberries and other fruits like peaches or grapes is very low—only 1–4 mg/kg raspberries (Larsen et al. 1991; Wang et al. 2019; Malik and Rawat 2021)—a direct isolation proves to be economically inefficient (Beekwilder et al. 2007; Wang et al. 2019). However, raspberry ketone precursors can be found in vegetables like rhubarb (Deifel 1989) or in the bark of plants like birch (*Betula* spp. (Santamour and Vettel 1978; Santamour and Lundgren 1997; Liimatainen et al. 2012)), rhododendron (*Rhododendron* spp. (Thieme et al. 1969; Parmar and Tripathi 1991)), or yews (*Taxus* spp. (Parmar and Tripathi 1991; Fronza et al. 1999)) in significantly larger quantities, e.g., 24.5 g/kg in dried inner bark of *Betula pendula* (Liimatainen et al. 2012). The most abundant natural raspberry ketone precursor is the corresponding alcohol rhododendrol (4-(4-hydroxyphenyl)-butan-2-ol). Rhododendrol occurs as aglycone moiety of different glycosides with varying rhododendrol stereoisomers such as (epi)rhododendrin, apiosyl(epi)rhododendrin, or arabinosyl(epi)rhododendrin (Šmite et al. 1993; Santamour and Lundgren 1997). Even among the same genus, the contents of (*R*)- and (*S*)-rhododendrol can vary significantly: for example, in *Betula nana*, the (*R*)-enantiomer dominates with 97 %, whereas in *Betula fruticosa*, both enantiomers occur in nearly equal amounts, and in *Betula saposhnikovii*, glycosides with the (*S*)-enantiomer can be found predominantly (Falconnier et al. 1999).

In literature, different routes for the natural production of raspberry ketone are stated: on the one hand, this can be achieved by heterologous pathways in microorganisms incorporated via metabolic engineering. Approaches with engineered microorganisms like *E. coli*, *S. cerevisiae*, or *C. glutamicum* yielded product titers between 5 and 9.89 g/l raspberry ketone either starting from expensive *p*-coumaric acid (Beekwilder et al. 2007; Lee et al. 2016; Wang et al. 2019; Milke et al. 2020; Paulino 2021), lower-priced tyrosine (Farwick et al. 2019), 4-hydroxybenzylidene acetone (Yang et al. 2021), or fatty acids as alternative low-cost feedstock (Chang and Liu 2021). Furthermore, the *de novo* production of raspberry ketone was achieved by genetically modified *E. coli* or *C. glutamicum* strains that produce tyrosine by themselves yielding 19 mg/l (Cankar et al. 2019) or even up to 780 mg/l raspberry ketone (Schloesser and Lambert 2018). A drawback of these previously described methods is that “foods that […] contain ingredients produced from GMOs” have to be labeled as, e.g., “genetically modified” according to EU law (European Parliament 2003; Deckers et al. 2020), which might be deterrent to consumers.

On the other hand, strategies utilizing precursors like rhododendrol glycosides isolated from *Betulaceae* or other plants as starting material are described. The conversion of rhododendrol glycosides isolated from *B. alba* was attained by a commercial β-glucosidase and *Candida boidinii* cells providing alcohol dehydrogenase (ADH) activity with a maximum yield of 44.5 % (Dumont et al. 1996). A similar approach with yeast cells comprising both β-glucosidase and ADH activity resulted in 82.1 % raspberry ketone (Falconnier et al. 1999). Additionally, the kinetic resolution of racemic rhododendrol was demonstrated by using lyophilized cells of *Rhodococcus* spp. resulting in a conversion of 52 % (Kosjek et al. 2003). These procedures have in common that only one of the rhododendrol enantiomers is converted leaving space for improvement depending on the composition of the starting material.

**Fig. 1:**
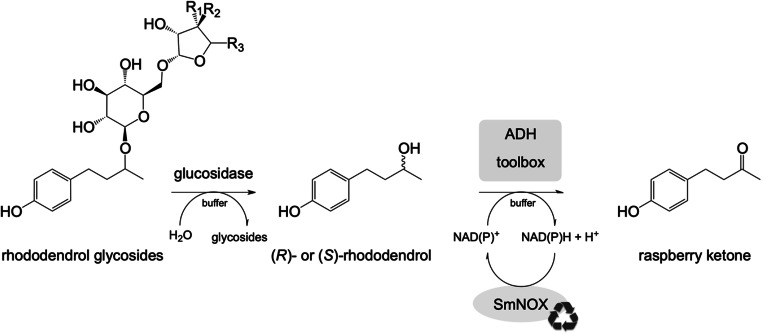
Reaction scheme for the natural production of raspberry ketone starting from naturally occurring rhododendrol glycosides. Rhododendrol glycosides are hydrolyzed by a glucosidase followed by the oxidation of the intermediates (*R*)- and (*S*)-rhododendrol to raspberry ketone by an ADH toolbox and a universal cofactor-regenerating oxidase SmNOX (Petschacher et al. 2014)

With this work, we overcame the limitations of existing strategies starting from rhododendrol glycosides by providing an ADH toolbox implemented in a biocatalytic cascade (Fig. 1). This toolbox contains ADHs with different selectivities for (*R*)- or (*S*)-rhododendrol and, additionally, a universal cofactor regeneration system, thus, enabling the conversion of both rhododendrol enantiomers from various starting materials isolated from natural resources to achieve a higher conversion.

## Materials and methods

### Material

The substrate (1:1 rhododendrol glycoside mixture of arabinosyl- and apiosylrhododendrin derived from *Betula pendula*) and intermediate (racemic rhododendrol, NP-000438) were provided by AnalytiCon Discovery GmbH (Potsdam, DE). Raspberry ketone standard (68524) and commercial β-glucosidase from almonds (49290, 7.3 U/mg) were purchased from Sigma-Aldrich (St. Louis, USA). Chemically competent *E. coli* BL21(DE3) were obtained from New England Biolabs GmbH (Frankfurt am Main, DE) and *E. coli* C43(DE3) from Sigma-Aldrich (St. Louis, USA). ADH from *Equus caballus* (ADH1E, 142±27 U/l) codon-optimized and subcloned in pET-28a (GenBank: MW808988) and *Streptococcus mutans* NAD(P)H oxidase variant 193R194H (SmNOX, 28.8 U/ml) by Petschacher et al. 2014 codon-optimized and subcloned in pET-28a (GenBank: MW808989) were ordered at BioCat GmbH (Heidelberg, DE). Moreover, the following ADHs were used in this study: ADH from *Lactobacillus brevis* (LB-ADH, 17.2±0.1 U/ml, GenBank: MW808993) subcloned in pEG180 (originally provided by Prof. W. Kroutil, University of Graz, Austria), *Lactobacillus kefir* (LK-ADH, 30.1±2.8 U/ml, GenBank: MW808990) subcloned in pET-22b (originally provided by Prof. W. Hummel, University of Düsseldorf, Germany), *Leifsonia* sp. (LS-ADH, 90.3±5.7 U/l, GenBank: MW808992) subcloned in pEG50 (originally provided by Prof. W. Kroutil, University of Graz, Austria), and *Rhodococcus ruber* (RR-ADH, 20.1±1.5 U/ml, GenBank: MW808991) subcloned in pKA1.

### Expression and determination of activity of the alcohol dehydrogenases

All ADHs were expressed in *E. coli* C43(DE3) (LB-ADH (Sattler et al. 2014)) or *E. coli* BL21(DE3) (all other ADHs). In brief, 50 ml (or 300 ml) terrific broth (TB) medium supplemented with 100 μg/ml ampicillin (LB-, LK-, LS-ADH) or 100 μg/ml chloramphenicol (RR-ADH) was inoculated with 1 % (v/v) of the overnight culture, grown at 37 °C and 180 rpm until an OD_600_ of 0.8 (LB-ADH), 0.5 (LK-ADH), 0.6 (LS-ADH), or 0.3 (RR-ADH). Additionally, the media was supplemented with 1 mM MgCl_2_ (LB-ADH and LK-ADH) or 1 mM ZnCl_2_ (RR-ADH). Protein expression was induced by addition of 0.4 μM anhydrotetracycline (LB-ADH), 1 mM IPTG (LK-ADH), 0.4 mM IPTG (LS-ADH), or 40 μM IPTG (RR-ADH). The cells were grown for 22 h at 20 °C (LB-, LK-, and RR-ADH) or 37 °C (LS-ADH). ADH1E was expressed in 50 ml TB auto-induction medium supplemented with 50 μg/ml kanamycin, 2 mM MgSO_4_, 0.2 x trace elements, and inoculated with 1 % overnight culture. Cells were grown at 37 °C and 180 rpm for 17 h.

The cells were harvested by centrifugation (10,000×*g*, 3 min, 4 °C), washed with 10 ml of 25 mM sodium phosphate buffer pH 8, and disrupted via sonication (50 % power, 6x 30 s with 30 s breaks) with the SONOPULS HD 2070 (BANDELIN electronic GmbH & Co. KG, Berlin, DE). The crude lysate, received after centrifugation at 10,000×*g* and 4 °C for 30 min, was used for further experiments.

Activity of the crude enzyme lysate was determined spectrophotometrically via a NAD(P)H assay at 25 °C. For this purpose, 20 μl enzyme lysate was mixed with 0.5 mM NAD(P)^+^, 39 mM buffer, and 1 mM racemic rhododendrol in acetonitrile (MeCN, 2.5 % v/v) in a total volume of 200 μl. NADH formation was quantified at 340 nm using the Infinite M200 PRO microplate reader (Tecan Group, Männedorf, CH). One unit of activity was defined as the amount of enzyme forming 1 μmol NAD(P)H per minute under assay conditions.

### Expression and determination of activity of the cofactor-recycling enzyme SmNOX

SmNOX was expressed in *E. coli* BL21 (DE3). Fifty milliliter TB auto-induction medium supplemented with 50 μg/ml kanamycin was inoculated with 1 % overnight culture, grown for 6 h at 37 °C and 180 rpm, and, finally, cooled down to 20 °C for further growth overnight. Cells were harvested by centrifugation (10,000×*g*, 3 min, 4 °C), washed with 10 ml 50 mM CHES buffer pH 9 and disrupted via sonication (50 % power, 4x 1 min with 1 min breaks) with the SONOPULS HD 2070 (BANDELIN electronic GmbH & Co. KG, Berlin, DE). The crude lysate, received after centrifugation at 10,000×*g* and 4 °C for 30 min, was used for further experiments.

Activity was determined spectrophotometrically via a NADH assay at 25 °C. For this purpose, 20 μl enzyme lysate was mixed with 45 mM CHES buffer pH 9 and 1 mM NADH in a total volume of 200 μl. NADH consumption was quantified at 340 nm during 5 min using the Infinite M200 PRO microplate reader (Tecan Group, Männedorf, CH). One unit of activity was defined as the amount of enzyme consuming 1 μmol NADH per minute under assay conditions.

### Oxidation of rhododendrol by the alcohol dehydrogenases

One millimolar racemic rhododendrol (added from a stock solution prepared in MeCN, leading to a final concentration of 1 % MeCN in the reaction mixture) was oxidized in a buffered system by the addition of 200 μl recombinantly expressed ADH lysate (RR-ADH, ADH1E, LB-ADH, LK-ADH, or LS-ADH) in a total reaction volume of 500 μl in glass vials. One hundred micromolars of NAD(P)^+^ were added and recycled by 0.6 U/ml SmNOX. The reactions were performed in triplicates either in 28 mM CHES buffer pH 9 at 25 °C (RR-ADH and LB-ADH), 28 mM CHES buffer pH 9 at 40 °C (LK-ADH), or 28 mM glycine-NaOH buffer pH 10 at 40 °C (ADH1E and LS-ADH) at 1000 rpm in a ThermoMixer C (Eppendorf AG, Hamburg, DE).

Conversion of both rhododendrol enantiomers was achieved by applying 100 μl enzyme lysate of ADH1E and LK-ADH each. The reaction with 1 mM racemic rhododendrol in MeCN (1 %), 50 μM NAD^+^, 50 μM NADP^+^, and 0.6 U/ml SmNOX was conducted in triplicates in 28 mM glycine-NaOH buffer pH 10 at 40 °C and 1000 rpm in a ThermoMixer C (Eppendorf AG, Hamburg, DE).

### Enzymatic hydrolysis of rhododendrol glycosides

One to 10 mg/ml rhododendrol glycosides were hydrolyzed by 0.1 to 1 mg/ml ALM in glass vials and a reaction volume of 500 μl. The reaction took place in triplicates in 25 mM sodium acetate buffer pH 5.5 shaking at 40 °C and 1200 rpm in a ThermoMixer C (Eppendorf AG, Hamburg, DE).

### Two-step biocatalytic cascade for raspberry ketone production

One hundred fifty milligrams of the rhododendrol glycoside mix (21.7 mM) and 15 mg ALM (7.4 μM) were stirred in 15 ml 25 mM sodium acetate buffer pH 5.5 in a round-bottom flask at 40 °C for 24 h. The reaction was cooled down to 25 °C and diluted by a factor of 10 to a final volume of 150 ml by adding the following reactants: 50 ml LB-ADH lysate (5.7 U/ml), 100 μM NADP^+^, 0.6 U/ml SmNOX, and 55 mM CHES buffer pH 9. After 24 h, the reaction mixture was extracted six times with 50 ml ethyl acetate. The combined organic phases were evaporated to dryness and analyzed via GC and NMR.

### Analytics

MS measurements were performed using an expression^L^ Compact Mass Spectrometer with ESI ionization source (Advion, Ithaca, USA). The ^1^H NMR spectrum was recorded using a 400 MHz Avance Bruker spectrometer (Bruker Corporation, Billerica, USA). GC-FID analysis was conducted using a GC2010 (Shimadzu, Kyoto, JP) with a SolGel-WAX column (30 m × 0.25 mm × 0.25 μm; SGE Analytical Science, Melbourne, AU). One microliter sample was injected at 240 °C with the following column temperature program: 125 °C/4.5 min–10 °C/min–175 °C/0 min–25 °C/min–250 °C/9.5 min. Raspberry ketone: *T*_ret_=16.4 min, rhododendrol: *T*_ret_=17.2 min.

For HPLC analysis, 50 μl samples were extracted twice with 100 μl ethyl acetate. Combined organic phases were evaporated to dryness and resuspended in the corresponding HPLC solvent. Thereupon, samples were either analyzed by normal-phase HPLC (for separation of rhododendrol enantiomers) or reverse-phase HPLC (for analysis of rhododendrol glycosides). Normal-phase HPLC was performed on a VWR Hitachi LaChrom Elite system (VWR International, Radnor, USA) equipped with a Lux^®^ 5 μm Cellulose-1 column (250 × 4.6 mm; Phenomenex Inc., Torrance, USA). Ten microliter injected sample were separated at 30 °C in *n*-hexane/*i*-PrOH (9:1, v/v) at 0.8 ml/min isocratic flow and detected via UV at 220 nm. Reverse-phase HPLC was performed on a VWR Hitachi Chromaster system (VWR International, Radnor, USA) equipped with a Hypersil ODS 5 μm (250 ×4.6 mm) column (Agilent Technologies, Santa Clara, US). Ten microliter injected sample were separated at 40 °C in MeCN/ddH_2_O with 0.1 % formic acid (15:85, v/v) at 1 ml/min isocratic flow and detected via UV at 200 nm.

## Results

### Investigation of different alcohol dehydrogenases for the oxidation of rhododendrol

**Fig. 2:**
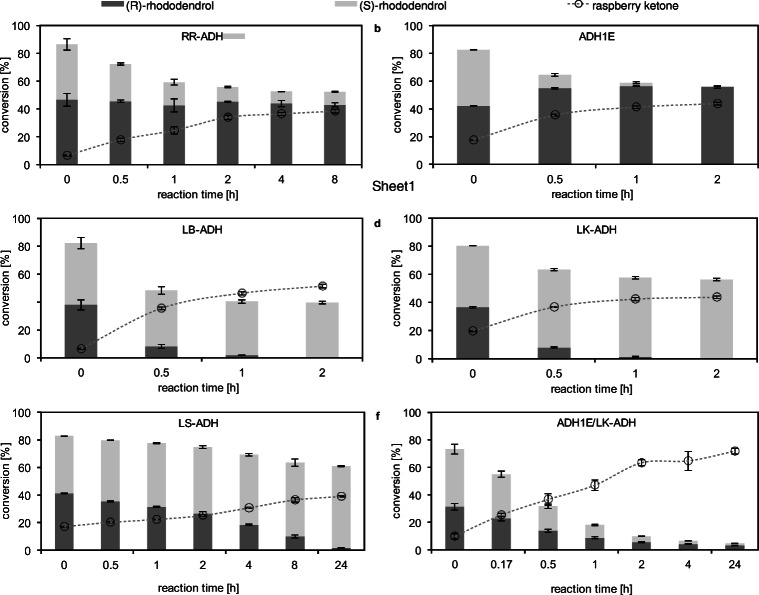
Time course of the oxidation of racemic rhododendrol (1 mM) to raspberry ketone catalyzed by **a** RR-ADH (8.0 U/ml), **b** ADH1E (57 U/l), **c** LB-ADH (6.9 U/ml), **d** LK-ADH (3.0 U/ml), **e** LS-ADH (36.1 U/l), and **f** a combination of ADH1E (28.5 U/l) and LK-ADH (6 U/ml)

In view of the various starting material with different rhododendrol enantiomers available in nature, different recombinantly expressed alcohol dehydrogenases (ADHs) were investigated for the oxidation of a racemic mixture of (*R*)- and (*S*)-rhododendrol to raspberry ketone. Out of the five investigated ADHs, enzymes converting either of the enantiomers of racemic rhododendrol could be identified (Table 1): the ADHs from *Rhodococcus ruber* (RR-ADH) and *Equus caballus* (ADH1E) both oxidized (*S*)-rhododendrol (Fig. 2a, b, S9 and S10), whereas ADHs from *Lactobacillus brevis* (LB-ADH), *Lactobacillus kefir* (LK-ADH), and *Leifsonia* sp. (LS-ADH) showed (*R*)-selectivity (Fig. 2c–e and Figure S11–13). The respective rhododendrol enantiomer was fully converted within two hours by ADH1E (Figure 2b), LB-ADH (Figure 2c) and LK-ADH (Figure 2d). With the same volume of LS-ADH lysate the oxidation proceeded considerably slower, full conversion of (*R*)-rhododendrol was only accomplished after 24 h. After 8 h reaction time with the RR-ADH 9.3±0.4 %, (*S*)-rhododendrol remained unconverted.

**Table 1:** Cofactor-dependency, enantioselectivity towards rhododendrol and conversion to raspberry ketone by the investigated ADHs in a certain reaction time (a maximum of 50 % conversion is theoretically possible)

ADH	Cofactor	Enantioselectivity	Conversion [%]	Reaction time [h]
RR-ADH (*Rhodococcus ruber*)	NAD^+^	(*S*)	38.4±1.4	8
ADH1E (*Equus caballus*)	NAD^+^	(*S*)	43.8±1.0	2
LB-ADH (*Lactobacillus brevis*)	NADP^+^	(*R*)	51.4±1.3	2
LK-ADH (*Lactobacillus kefir*)	NADP^+^	(*R*)	43.7±0.9	2
LS-ADH (*Leifsonia* sp.)	NAD^+^	(*R*)	39.0±0.4	24

By combining an (*S*)- and an (*R*)-selective ADH, such as ADH1E and LK-ADH, high conversion of both rhododendrol enantiomers was attained within 24 h resulting in 71.8±2.1 % raspberry ketone (Fig. 2f and Figure S14). During all these experiments, a cofactor recycling was successfully achieved by an engineered NAD(P)H oxidase from *Streptococcus mutans* (SmNOX) that oxidizes both NADH and NADPH with similar efficiency (Petschacher et al. 2014).

### Two-step biocatalytic cascade for raspberry ketone production

**Fig. 3:**
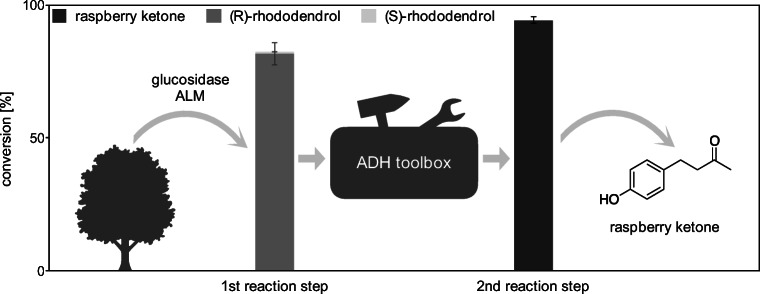
Schematic depiction of the biocatalytic cascade for raspberry ketone production including achieved conversions for each reaction step

Following, these rhododendrol converting ADHs were aimed to be applied as a second step in a biocatalytic cascade with rhododendrol glycosides as starting material (Figs. 1 and 3). In this case, a 1:1 mixture of mainly arabinosyl- and apiosylrhododendrin originating from *Betula pendula* was used.

The first reaction step, the hydrolysis of the glycosidic bond of the rhododendrol glycosides to release the raspberry ketone precursor rhododendrol, was accomplished by a commercial β-glucosidase from almonds (ALM) showing highest activity at pH 5.5 and at least 40 °C (Becker et al. 2020). The influence of the substrate load was investigated in concentrations up to 10 mg/ml (~21.7 mM) resulting in a conversion of 80±1% within only 2 h using 1 mg/ml (~7.4 μM) ALM in small-scale reactions (Figures S15-S17). The formed rhododendrol predominantly showed (*R*)-configuration (99.1 % HPLC peak area; Figure S18).

After these optimizations of the first reaction step, a two-step biocatalytic cascade with 150 mg of the rhododendrol glycoside mixture was conducted (Fig. 3). The hydrolysis of the rhododendrol glycosides (10 mg/ml) by ALM (1 mg/ml) showed 82±4 % conversion after 24 h (Fig. 3 and Figure S19). As the hydrolysis of the rhododendrol glycosides predominantly resulted in (*R*)-rhododendrol, the (*R*)-specific LB-ADH (5.7 U/ml) was applied in the second reaction step of the cascade together with the cofactor-recycling enzyme SmNOX. After 24 h, 94±1% of the rhododendrol was converted while, additionally, remaining rhododendrol glycosides from the first reaction step were even further hydrolyzed during this second reaction step (Figure S20). In total, 55 mg raspberry ketone (m/z 163 [M-H]^-^) were isolated by extraction in a purity of approximately 90 % (determined via NMR analysis, Figures S1 and S2, Table S1; 91 % purity according to GC analysis, Figure S23) starting from 150 mg raw material. This corresponds to an isolated yield of approximately 93 %.

## Discussion

### Investigation of different alcohol dehydrogenases for the oxidation of rhododendrol

Five ADHs that convert either (*R*)- or (*S*)-rhododendrol are presented in this study. These ADHs showed the same selectivities concerning rhododendrol as described in literature for similar substrates (Kosjek et al. 2004; Inoue et al. 2005a; Weckbecker and Hummel 2006; Leuchs and Greiner 2011; Quaglia et al. 2012). All ADHs are compatible in terms of pH range (Figure S24) being preferably used between pH 9 and 10 for oxidation reactions (Kosjek et al. 2004; Inoue et al. 2005a; Quaglia et al. 2012). Moreover, reactions are ideally conducted between 25 and 40 °C.

Depending on the optical purity of the intermediate rhododendrol, one or two suitable ADHs can be chosen from the herein presented toolbox. We demonstrated this with a racemic mixture of rhododendrol that was converted to 71.8±2.1 % raspberry ketone by the combinatorial use of an (*R*)- and an (*S*)-selective ADH (LK-ADH and ADH1E). This conversion attained by using these two ADHs with contrary selectivity clearly outperforms the process presented in literature where only one enantiomer of racemic rhododendrol was converted yielding 52 % raspberry ketone (Kosjek et al. 2003).

The challenge concerning the recycling of expensive cofactors was elegantly solved by applying the engineered water-forming oxidase SmNOX that is capable of oxidizing both NADH and NADPH with similar efficiency (Petschacher et al. 2014). This proved to be particularly beneficial as the enzymes used in this study are dependent on different cofactors: (*R*)-selective LK- and LB-ADH use NADP^+^, whereas the remaining ADHs require NAD^+^ for catalysis (Table 1).

### Two-step biocatalytic cascade for raspberry ketone production

A 1:1 mixture of arabinosyl- and apiosylrhododendrin originally derived from *Betula pendula* was utilized as a starting material. The commercially available glucosidase ALM showed efficient conversion of this rhododendrol glycoside mixture to predominantly (*R*)-rhododendrol. Compared to literature this finding confirms that the (*R*)-enantiomer of rhododendrol dominates in rhododendrol glycoside material originating from *Betula pendula* (Šmite et al. 1993; Liimatainen et al. 2008; Liimatainen et al. 2012).

Hence, for the two-step biocatalytic cascade in a preparative scale using 150 mg rhododendrol glycosides, the (*R*)-selective LB-ADH was chosen from the toolbox for the second reaction step in view of the optical purity of the starting material. The enzymes LK-ADH and LS-ADH would have been suitable as well. This cascade reaction with the glucosidase ALM and the alcohol dehydrogenase LB-ADH yielded approximately 93 % isolated raspberry ketone which slightly surpasses the result achieved by Falconnier et al. 1999 who used a *Pichia* strain showing only activity towards (*R*)-rhododendrol. However, as they started from rhododendrol glycosides from *B. alba* where the (*R*)-rhododendrol dominates as well with 95 % (Falconnier et al. 1999), they could report a similar high conversion to 82.1 % raspberry ketone. In contrast to this, with our ADH toolbox including SmNOX for easy cofactor regeneration, we are able to convert a variety of starting materials with high yields as we can not only use rhododendrol glycosides with high proportions of (*R*)-rhododendrol but also (*S*)-rhododendrol by selecting different ADHs from our toolbox. Even the conversion of a mixture of starting materials from different natural sources without prior analysis of the optical purity would be possible by utilizing the ADH toolbox.

With this study, we lay a solid foundation for future process optimization studies where parameters like substrate load, the ratios of the applied ADHs, mass transfer, or downstream processing may be optimized to achieve an economically efficient scale-up procedure. It may also be considered to expand the toolbox with non-stereoselective ADHs, e.g., by protein engineering (Musa et al. 2015). Besides, the ADH toolbox along with the universal cofactor-recycling enzyme SmNOX might potentially also be used for the conversion of similar enantiomeric substrates, especially in view of the broad substrate spectra of the ADHs (Inoue et al. 2005b; Weckbecker and Hummel 2006; Leuchs and Greiner 2011; Hollmann et al. 2012; Rodríguez et al. 2014; Itoh 2014). Finally, this strategy benefits from being GMO-free compared to known raspberry production pathways in engineered microorganisms (e.g., Schloesser and Lambert 2018) resulting in a higher acceptance at the food market.

## Supplementary Information

ESM 1 (PDF 696 kb)
