# Supplementary material for: An ADH toolbox for raspberry ketone production from natural resources via a biocatalytic cascade
Source: Appl Microbiol Biotechnol. 2021 May 14;105(10):4189–97. doi: 10.1007/s00253-021-11332-9 (PMC8140976; doi:10.1007/s00253-021-11332-9)
Supplement: Supplementary file 1 — (PDF 696 kb) [file 253_2021_11332_MOESM1_ESM.pdf]

Supplementary Information

Applied Microbiology and Biotechnology

An ADH toolbox for raspberry ketone production from natural resources via a biocatalytic cascade

Aileen Becker[1] · Dominique Böttcher[1] · Werner Katzer[2] · Karsten Siems[2] · Lutz Müller-Kuhrt[2] · Uwe T. Bornscheuer[1*]

[1] Department of Biotechnology and Enzyme Catalysis, Institute of Biochemistry, University of Greifswald, Greifswald, Germany

[2] AnalytiCon Discovery GmbH, Potsdam, Germany

*E-mail: uwe.bornscheuer@uni-greifswald.de

Table of contents

1. Additional experimental information……………………………………………… 3

1.1. NMR analysis……………………………………………………………………… 3

2. Supplementary figures………………………………………………………………… 5

2.1. Standard curves for quantification of HPLC analysis…………………………… 5

2.2. Results: Investigation of different ADHs for the oxidation of rhododendrol…… 7

2.3. Results: Two-step biocatalytic cascade for raspberry ketone production………. 10

2.4. Miscellaneous…………………………………………………………………… 15

3. Sequences…………………………………………………………………………… 16

1. Additional experimental information

1.1. NMR analysis

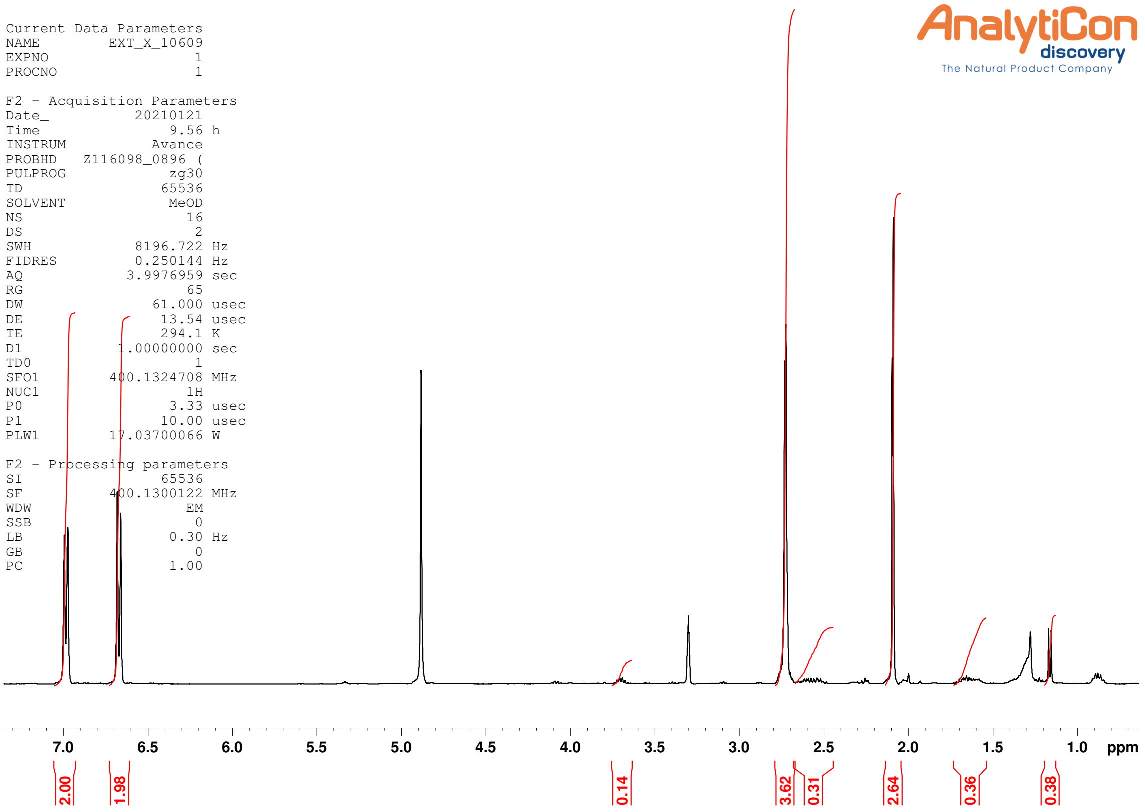

Figure S1 ^{1}H-NMR spectrum at 400 MHz in methanol-d_4 of the isolated product, raspberry ketone, obtained by the two-step biocatalytic cascade starting from rhododendrol glycosides. The signals at 1.16 ppm (3H. 7 Hz, H-10) and 3.70 ppm (1H, tq, J = 7 and 7 Hz, H9) correspond to the intermediate rhododendrol, resulting in a purity of approx. 90 % for the final product raspberry ketone.

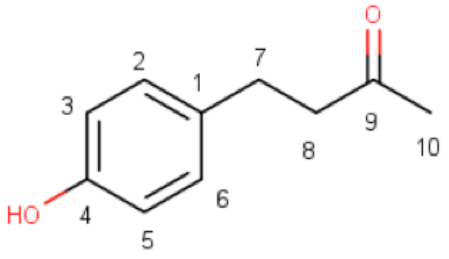

Figure S2 Structure of the product, raspberry ketone, with labeled protons.

Table S1 Protons of figure S2 assigned to the ^{1}H-NMR signals of figure S1 including multiplicity.

<table>
<tr><th>Proton</th><th>Shift [ppm]</th><th>Multiplicity, J [Hz]</th></tr>
<tr><td>2 = 6</td><td>6.98 (2H)</td><td>AB - System</td></tr>
<tr><td>3 = 5</td><td>6.75 (2H)</td><td>AB - System</td></tr>
<tr><td>7</td><td rowspan="2">2.73 (4H)</td><td rowspan="2">m</td></tr>
<tr><td>8</td></tr>
<tr><td>10</td><td>2.08 (3H)</td><td>s</td></tr>
</table>

2. Supplementary figures

2.1. Standard curves for quantification of HPLC analysis

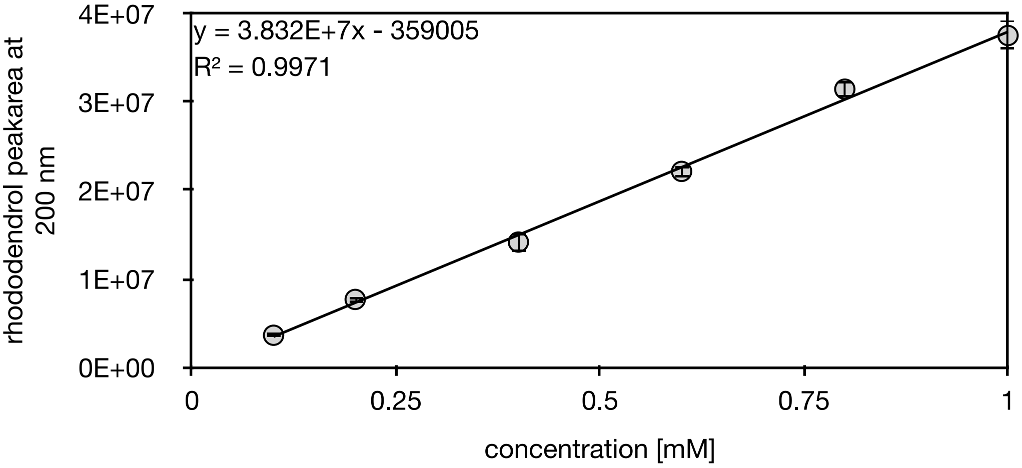

Figure S3 Rhododendrol standard curve analyzed via reverse phase HPLC equipped with a UV detector (200 nm). Each concentration was measured in triplicate.

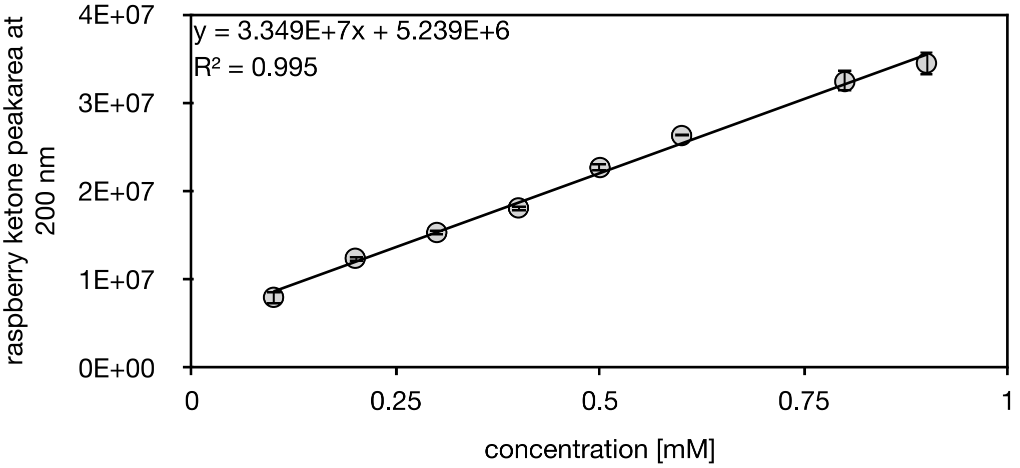

Figure S4 Raspberry ketone standard curve analyzed via reverse phase HPLC equipped with a UV detector (200 nm). Each concentration was measured in triplicate.

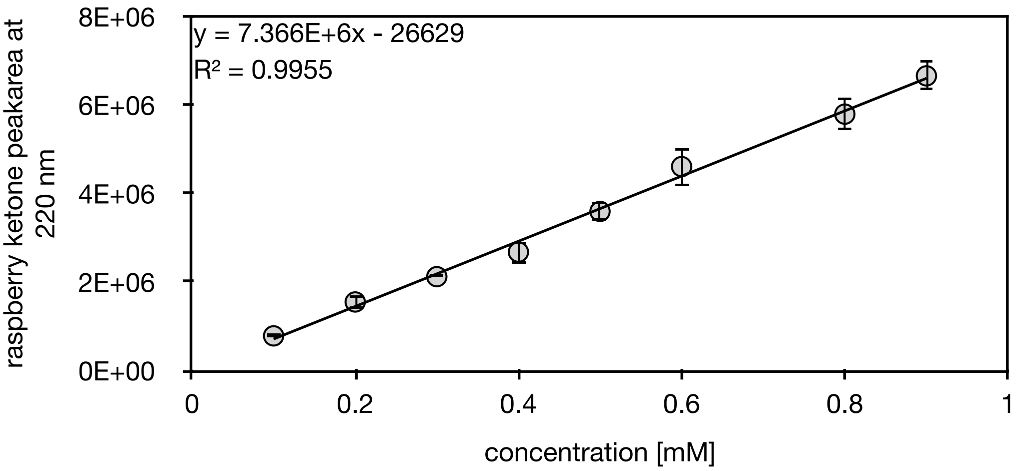

Figure S5 Raspberry ketone standard curve analyzed via normal phase HPLC equipped with a UV detector (220 nm). Each concentration was measured in triplicate.

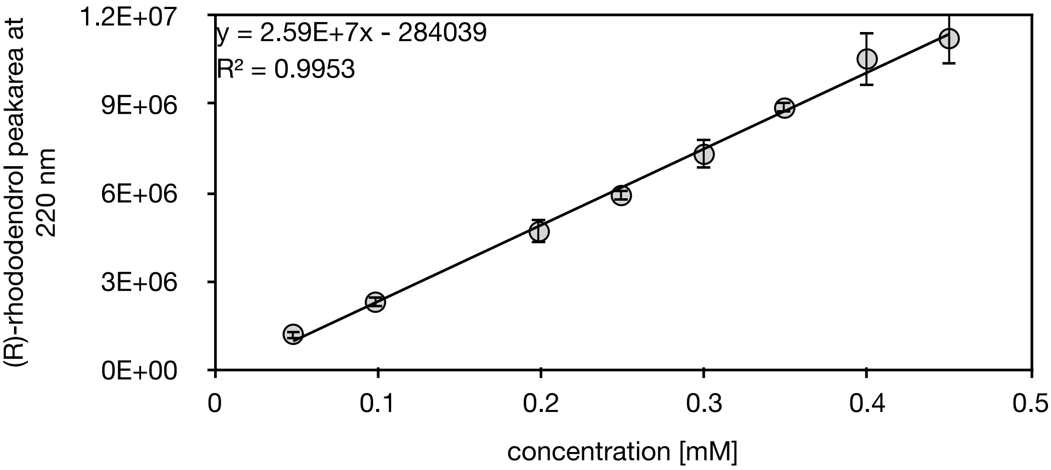

Figure S6 (*R*)-Rhododendrol standard curve analyzed via normal phase HPLC equipped with a UV detector (220 nm). Each concentration was measured in triplicate.

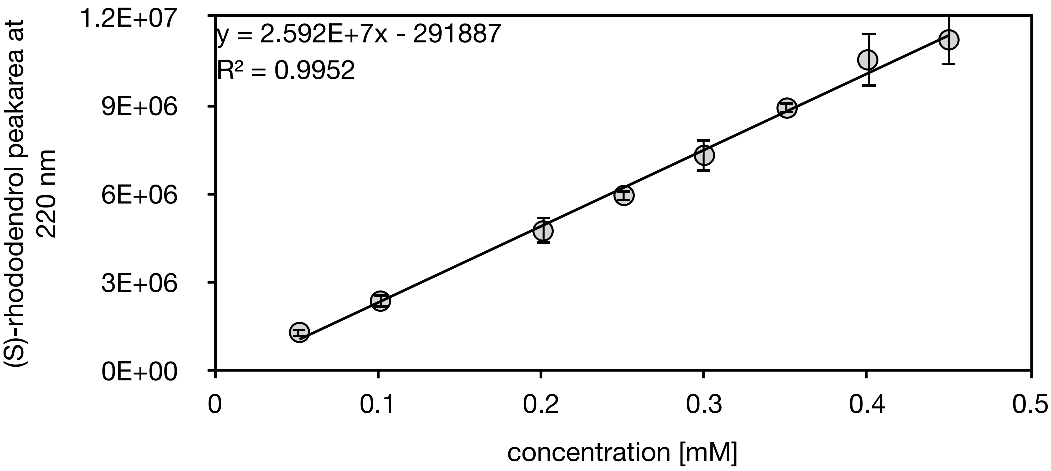

Figure S7 (*S*)-Rhododendrol standard curve analyzed via normal phase HPLC equipped with a UV detector (220 nm). Each concentration was measured in triplicate.

2.2. Results: Investigation of different ADHs for the oxidation of rhododendrol

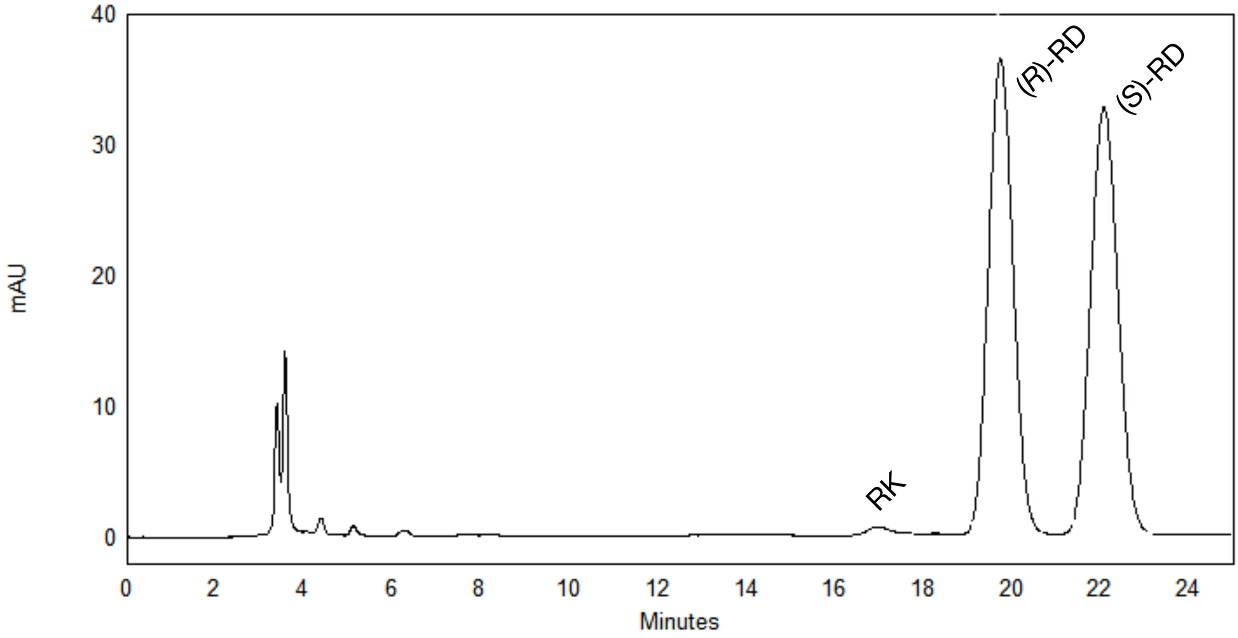

Figure S8 Chromatogram of normal phase HPLC analysis (220 nm) of the negative control of racemic rhododendrol (RD, 1 mM) without any enzyme at reaction start at 40 °C.

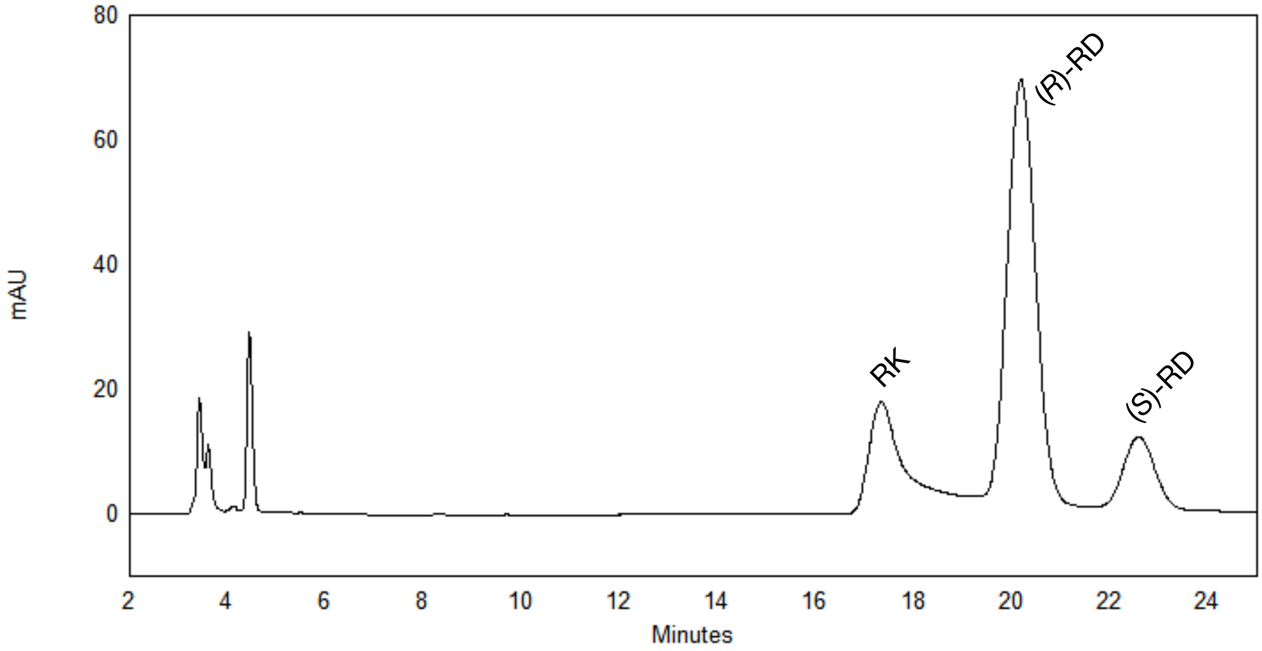

Figure S9 Chromatogram of normal phase HPLC analysis (220 nm) of the oxidation of racemic rhododendrol (RD, 1 mM) to raspberry ketone (RK) catalyzed by (*S*)-selective RR-ADH (8.0 U/ml) after 8 h reaction time at 25 °C.

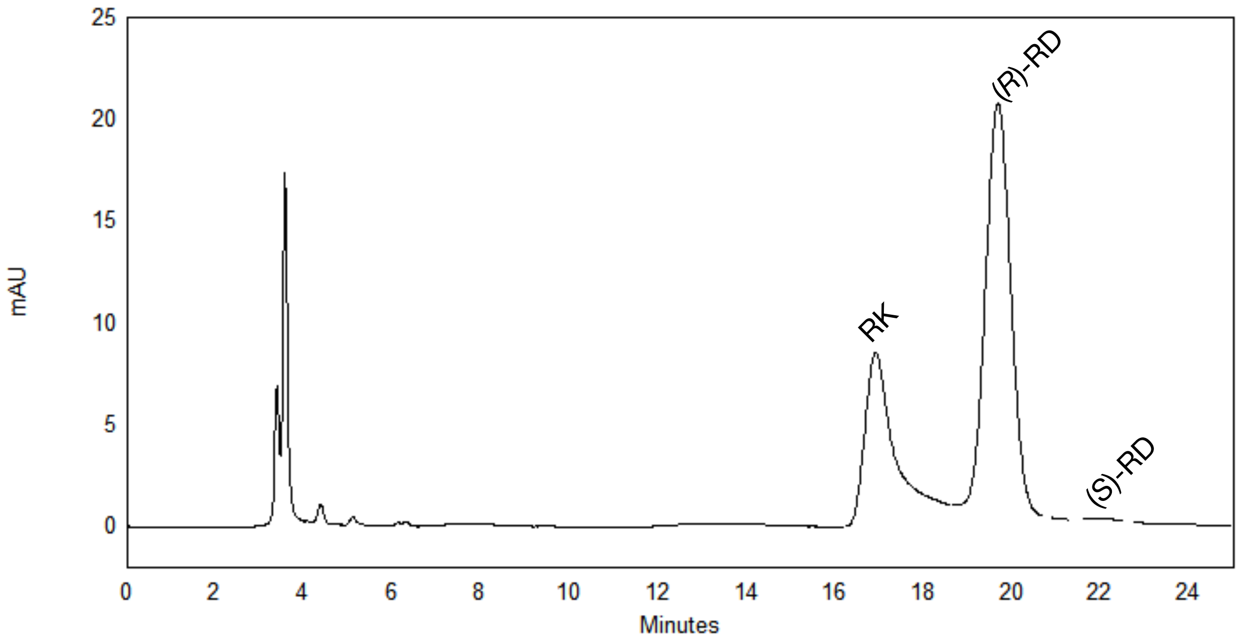

Figure S10 Chromatogram of normal phase HPLC analysis (220 nm) of the oxidation of racemic rhododendrol (RD, 1 mM) to raspberry ketone (RK) catalyzed by (*S*)-selective ADH1E (57 U/l) after 2 h reaction time at 40 °C.

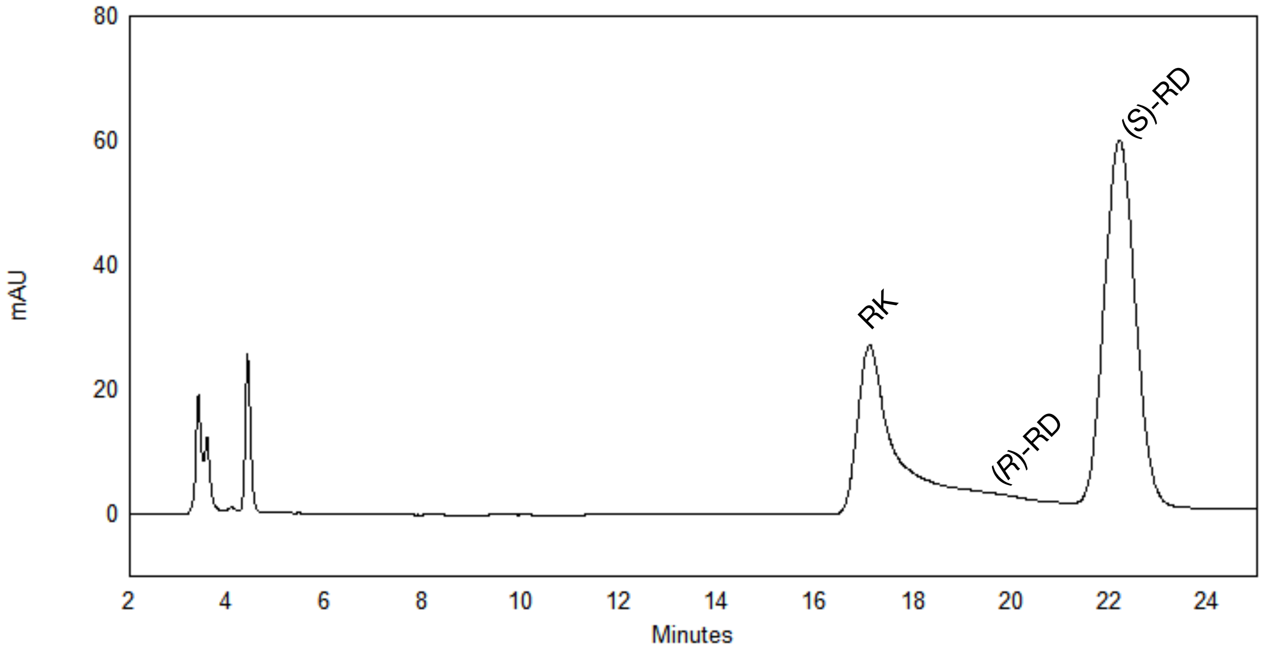

Figure S11 Chromatogram of normal phase HPLC analysis (220 nm) of the oxidation of racemic rhododendrol (RD, 1 mM) to raspberry ketone (RK) catalyzed by (*R*)-selective LB-ADH (6.9 U/ml) after 2 h reaction time at 25 °C.

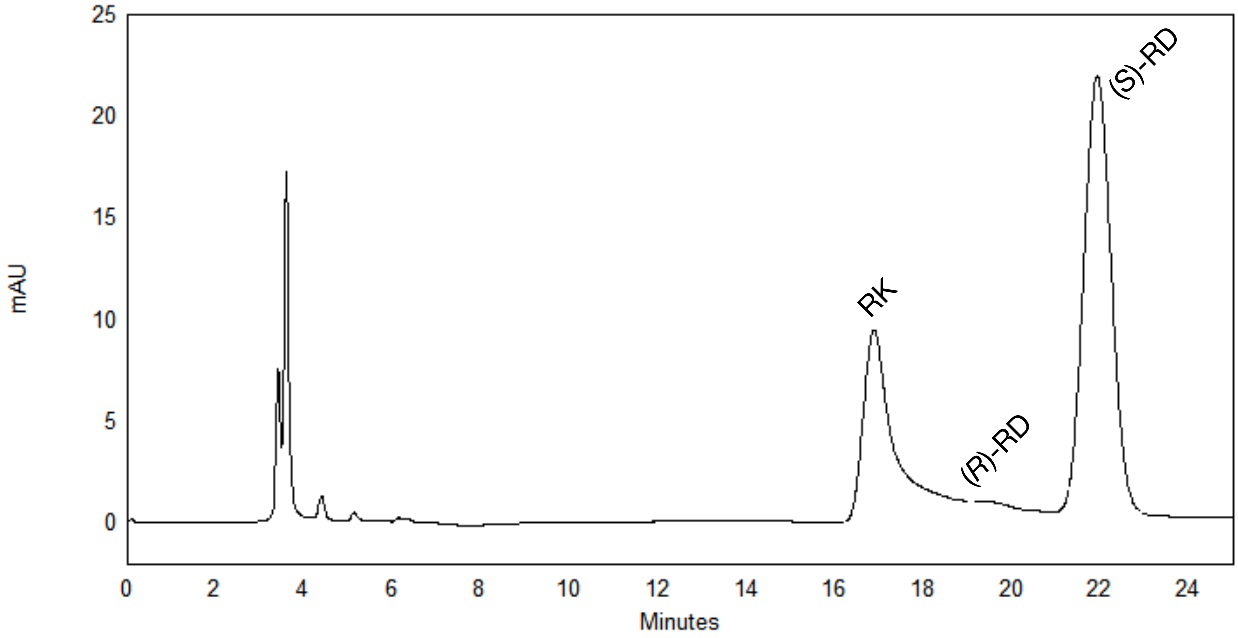

Figure S12 Chromatogram of normal phase HPLC analysis (220 nm) of the oxidation of racemic rhododendrol (RD, 1 mM) to raspberry ketone (RK) catalyzed by (*R*)-selective LK-ADH (3.0 U/ml) after 2 h reaction time at 40 °C.

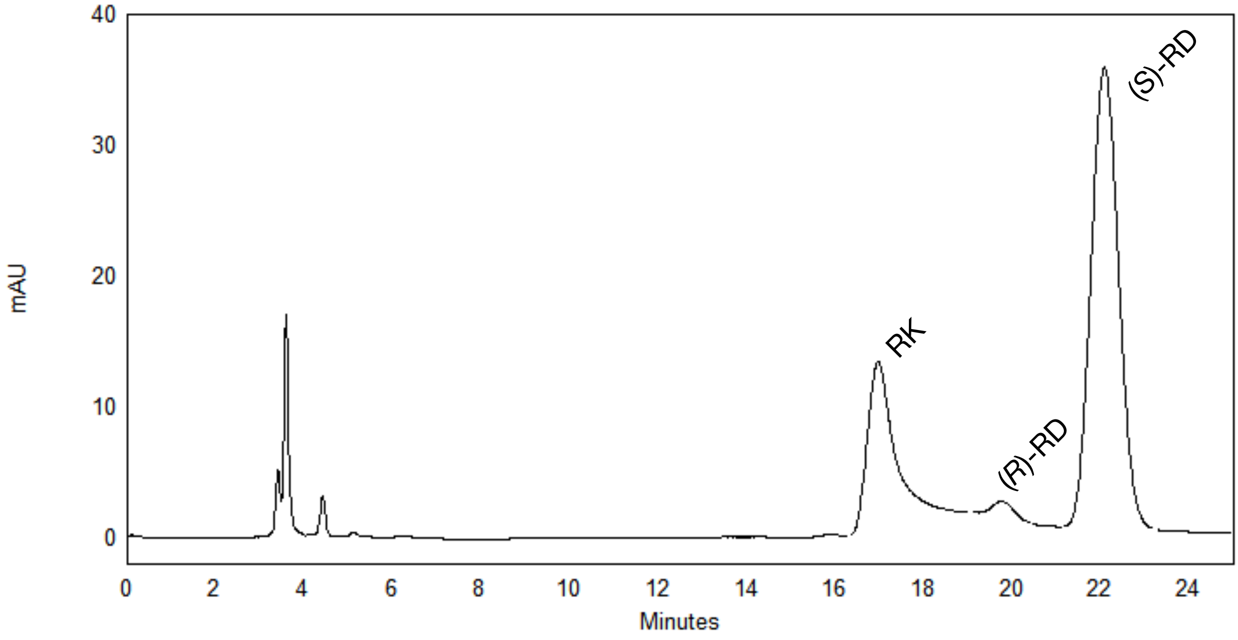

Figure S13 Chromatogram of normal phase HPLC analysis (220 nm) of the oxidation of racemic rhododendrol (RD, 1 mM) to raspberry ketone (RK) catalyzed by (*R*)-selective LS-ADH (36.1 U/l) after 24 h reaction time at 40 °C.

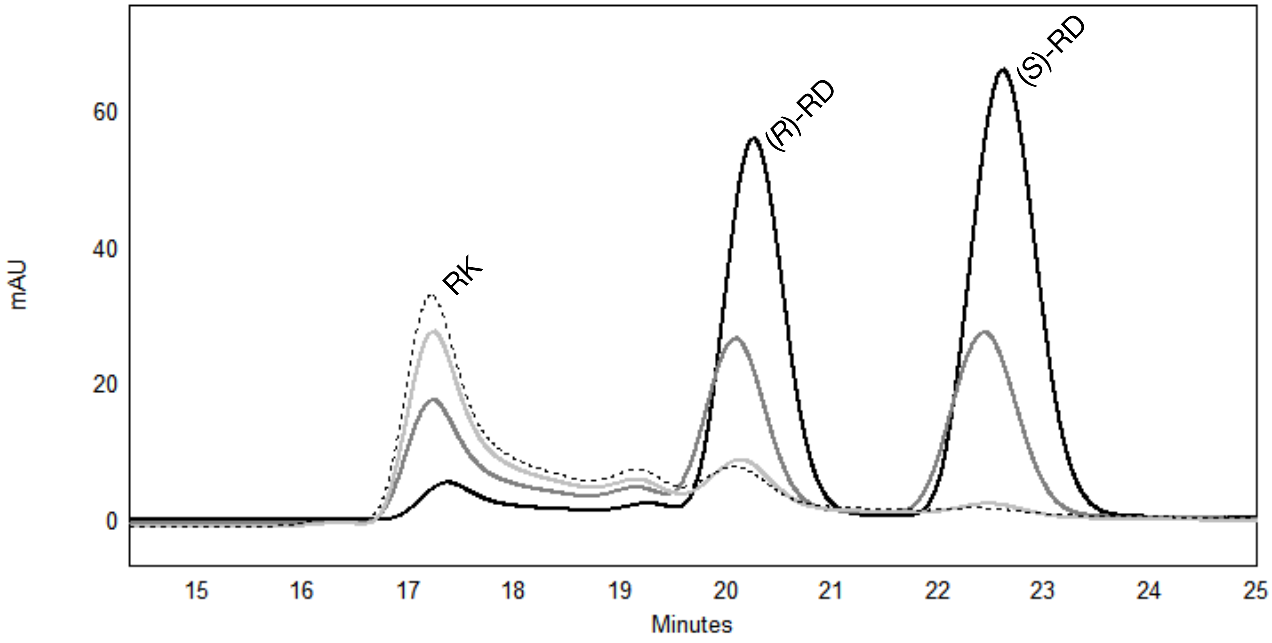

Figure S14 Clipped chromatogram of normal phase HPLC analysis (220 nm) of the oxidation of racemic rhododendrol (RD, 1 mM) to raspberry ketone (RK) catalyzed by the combination of (*S*)-selective ADH1E (28.4 U/l) and (*R*)-selective LK-ADH (6.0 U/ml) after 0 h (black), 0.5 h (dark grey), 4 h (light grey), and 24 h (black dots) reaction time at 40 °C.

2.3. Results: Two-step biocatalytic cascade for raspberry ketone production

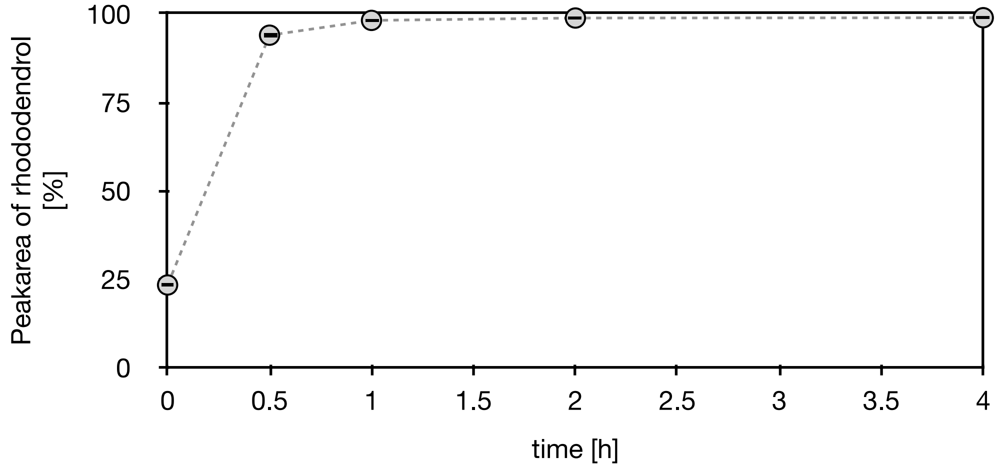

Figure S15 Time course of rhododendrol glycoside (1 mg/ml) hydrolysis catalyzed by the glucosidase from almonds (ALM, 0.1 mg/ml) at 40 °C. Glycoside hydrolysis is represented by the percentage of rhododendrol peakarea based on the total peakarea of both rhododendrol glycosides and rhododendrol. Maximum conversion is achieved after one hour.

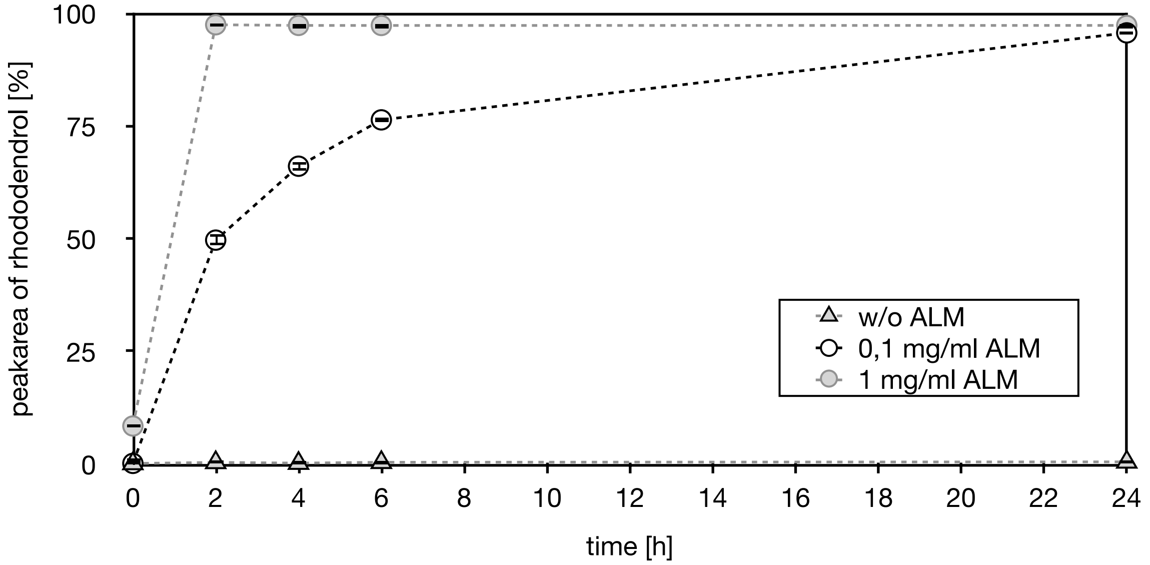

Figure S16 Time course of rhododendrol glycoside (10 mg/ml) hydrolysis catalyzed by different concentrations of the glucosidase ALM (0-1 mg/ml) at 40 °C. Glycoside hydrolysis is represented by the percentage of rhododendrol peak area based on the total peak area of both rhododendrol glycosides and rhododendrol.

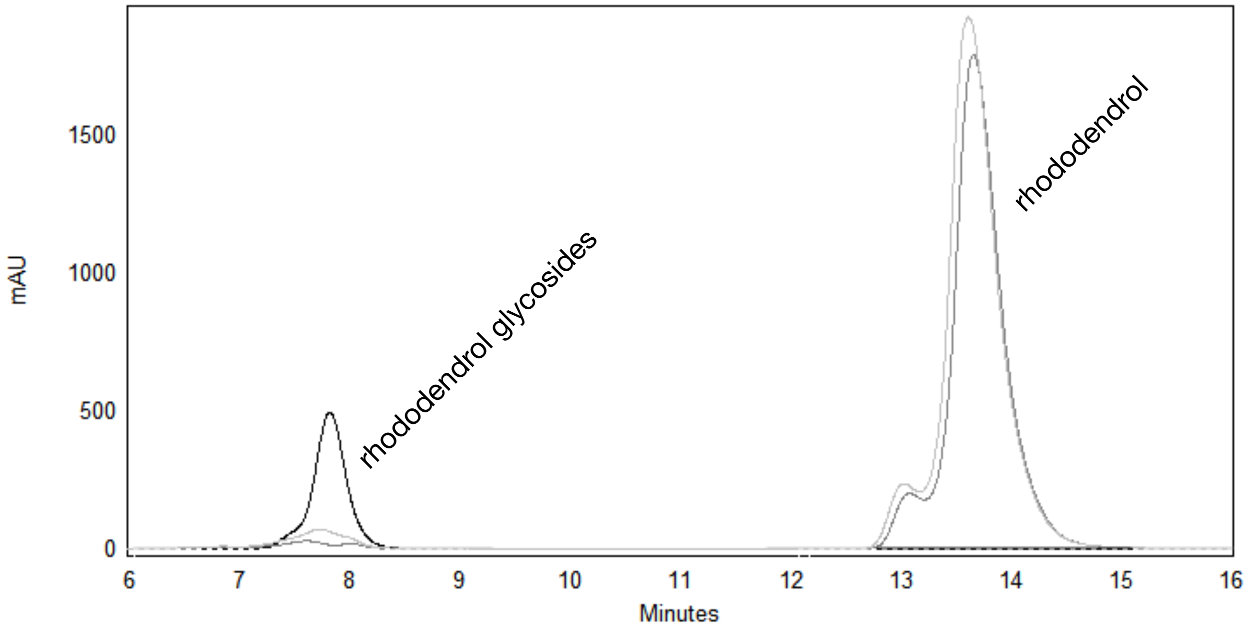

Figure S17 Chromatogram of reverse phase HPLC analysis (200 nm) of the hydrolysis of rhododendrol glycoside mixture (RG) catalyzed by the glucosidase ALM at different concentrations and time points. Black: negative control without enzyme at reaction start; dark grey: 10 mg/ml RG and 1 mg/ml ALM after 2 h; light grey: 10 mg/ml RG and 0.1 mg/ml ALM after 24 h.

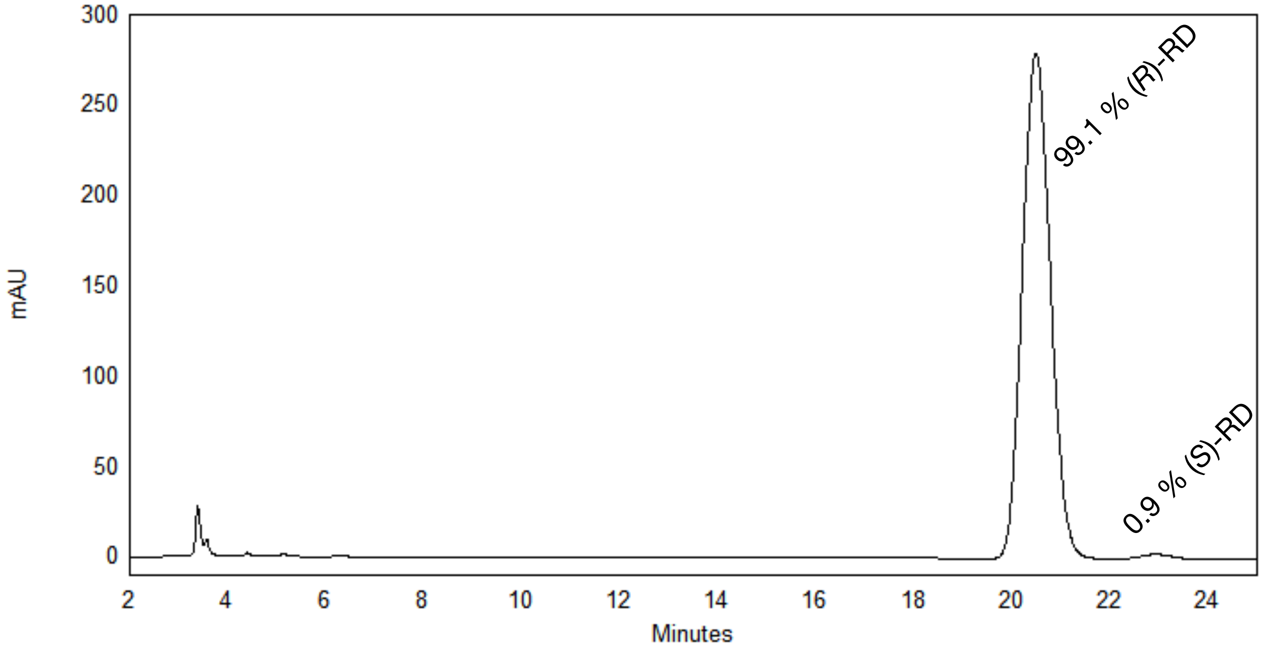

Figure S18 Chromatogram of normal phase HPLC analysis (220 nm) of the optical purity of the formed rhododendrol via hydrolysis of rhododendrol glycoside mixture catalyzed by the glucosidase ALM after 4 h reaction time at 40 °C.

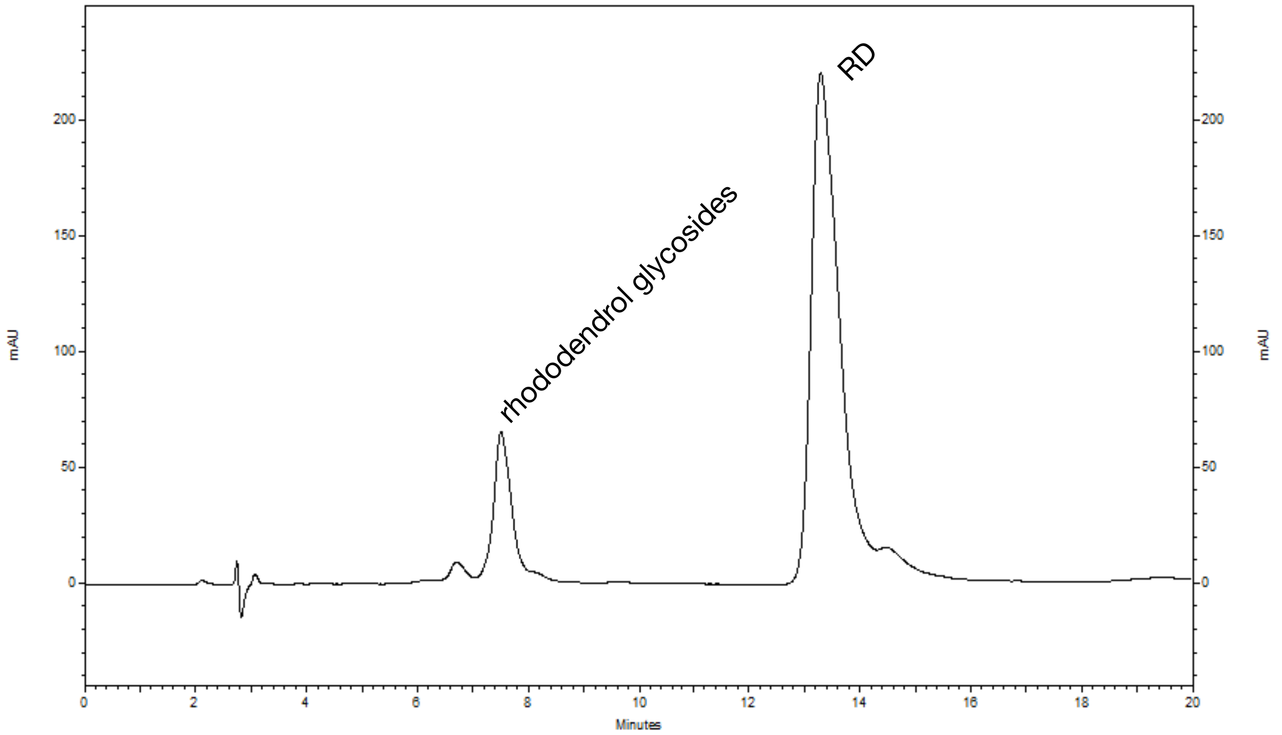

Figure S19 Chromatogram of reverse phase HPLC analysis (200 nm) of the first reaction step of the 2-step biocatalytic cascade starting from 150 mg rhododendrol glycosides after 24 h reaction time at 40 °C. The hydrolysis of rhododendrol glycosides (10 mg/ml) to rhododendrol (RD) is catalyzed by the glucosidase ALM (1 mg/ml).

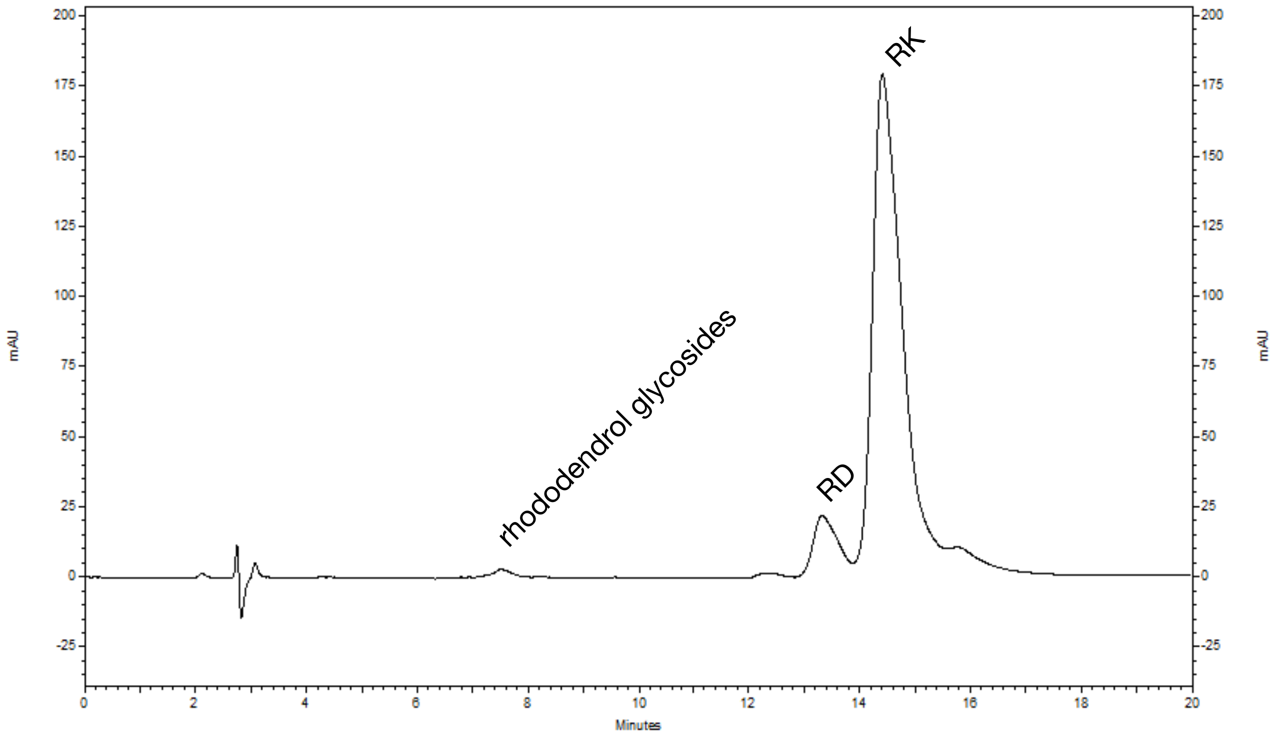

Figure S20 Chromatogram of reverse phase HPLC analysis (200 nm) of the second reaction step of the 2-step biocatalytic cascade starting from 150 mg rhododendrol glycosides after 24 h reaction time at 25 °C. The oxidation of the intermediate rhododendrol (RD) to raspberry ketone (RK) is catalyzed by LB-ADH (1 mg/ml).

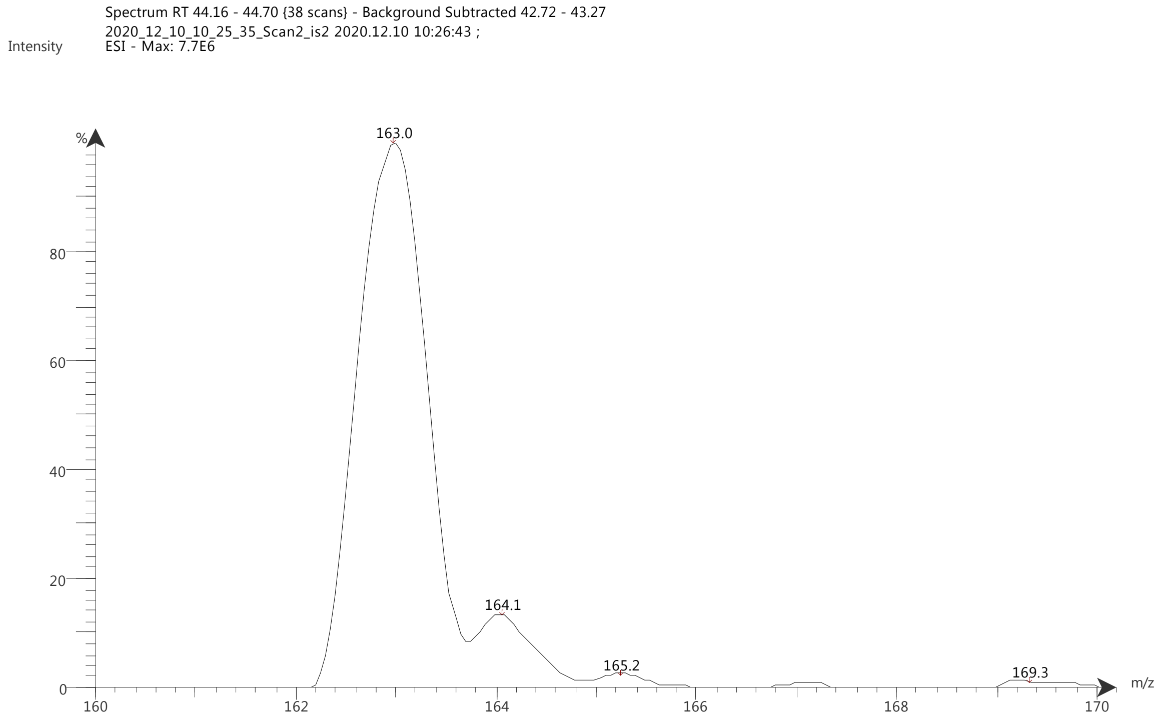

Figure S21 Chromatogram of ESI-MS analysis of the product of the second reaction step of the 2-step biocatalytic cascade starting from 150 mg rhododendrol glycosides after 24 h reaction time. Measurement was conducted with *expression*^{L} *Compact Mass Spectrometer* with ESI ionization source (Advion Inc., Ithaca, US) coupled to *Plate Express* (Advion Inc., Ithaca, US).

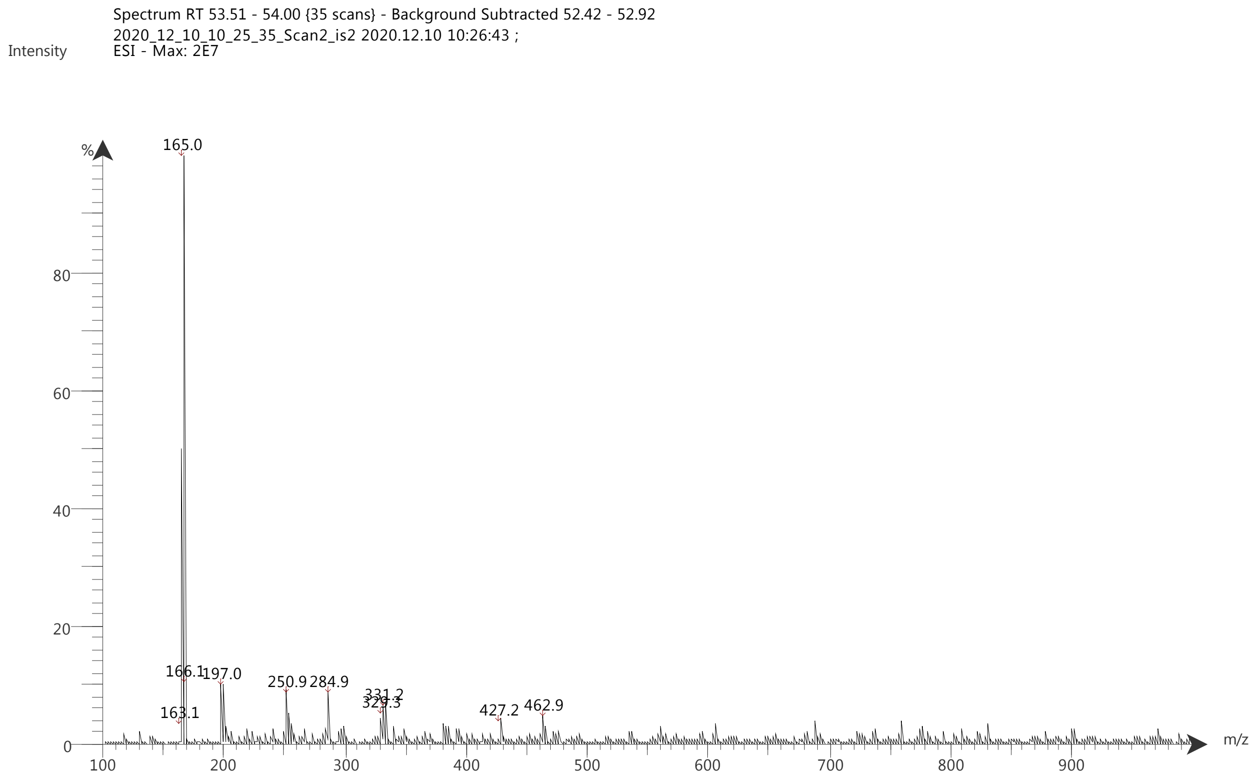

Figure S22 Chromatogram of ESI-MS analysis of racemic rhododendrol. Measurement was conducted with *expression*^{L} *Compact Mass Spectrometer* with ESI ionization source (Advion Inc., Ithaca, US) coupled to *Plate Express* (Advion Inc., Ithaca, US).

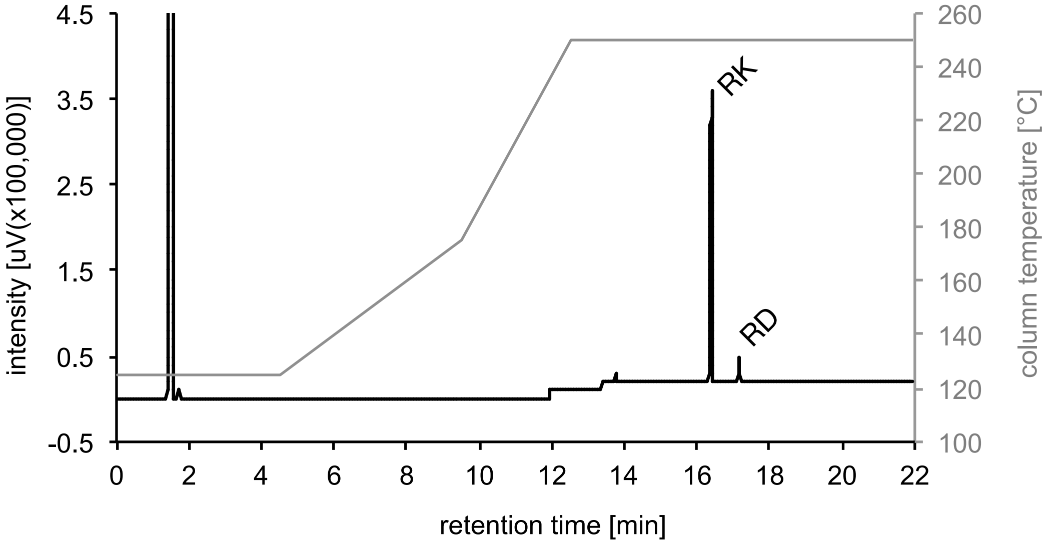

Figure S23 Chromatogram of GC analysis of the extracted end product (~5 mM) of the 2-step biocatalytic cascade for obtaining raspberry ketone starting from 150 mg rhododendrol glycosides. Raspberry ketone (RK): T_{ret}=16.4 min, rhododendrol (RD): T_{ret}=17.2 min.

2.4. Miscellaneous

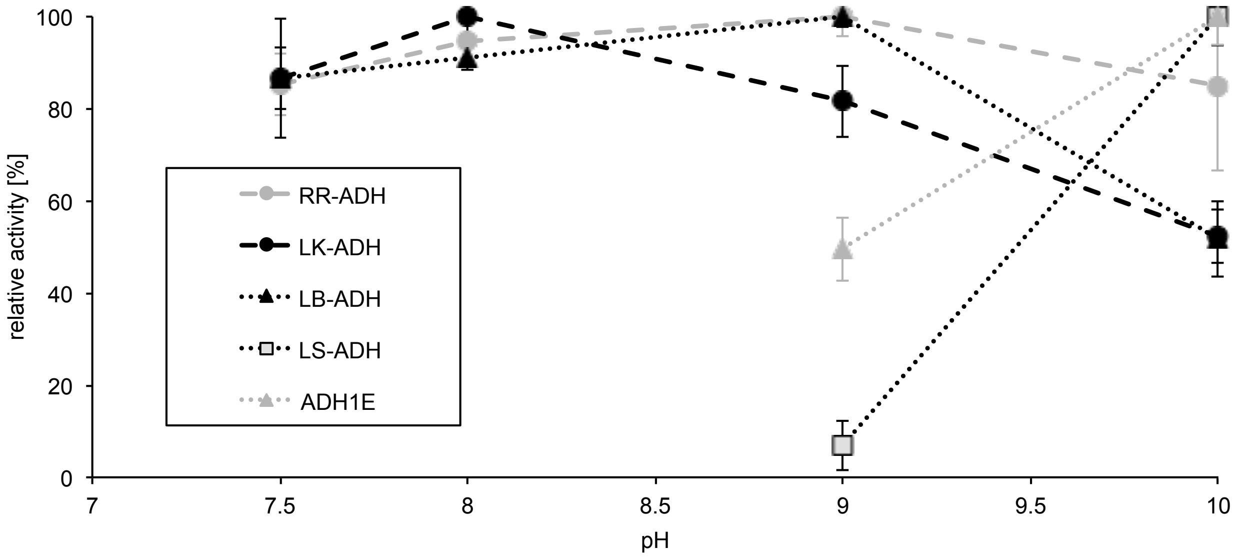

Figure S24 Relative activity of alcohol dehydrogenases at different pH values and buffer systems determined via NAD(P)H (0.5 mM) assay with rhododendrol (1 mM, 2.5 % ACN) as substrate at 25 °C. Used buffers: TRIS-HCl pH 7.5, sodium phosphate pH 8, CHES pH 9, Glycine-NaOH pH 10.

3. Sequences

DNA sequence of ADH1E

ATGAGCACCGCCGGTAAAGTGATTAAATGCAAAGCCGCCGTTCTGTGGGAAGAAAAAAAACCGTTCAGC
ATTGAAGAAGTTGAAGTTGCACCGCCGAAAGCCCATGAAGTGCGTATTAAAATGGTGGCAACCGGCATC
TGTCGTAGTGATGATCATGTTGTTAGCGGCACCCTGGTTACCCCGCTGCCGGTTATTGCCGGTCATGAAG
CCGCAGGCATTGTGGAAAGCATTGGCGAAGGTGTGACCACCGTGCGCCCGGGTGATAAAGTTATTCCG
CTGTTCACCCCGCAGTGCGGCAAATGTCGTGTGTGTAAACATCCGGAAGGTAACTTCTGTCTGAAAAATG
ATCTGAGCATGCCGCGTGGTACCATGCAGGATGGCACCAGCCGCTTCACCTGCCGCGGTAAACCGATTC
ATCACTTCCTGGGCACCAGCACCTTCAGCCAGTATACCGTTGTGGATGAAATTAGTGTTGCAAAAATTGAT
GCAGCCAGCCCGCTGGAAAAAGTGTGTCTGATTGGTTGTGGCTTCAGCACCGGTTATGGTAGTGCCGT
GAAAGTGGCCAAAGTGACCCAGGGCAGCACCTGTGCCGTGTTCGGCCTGGGCGGTGTTGGTCTGAGT
GTTATTATGGGTTGCAAAGCCGCGGGTGCCGCCCGTATTATTGGTGTGGATATTAATAAAGACAAGTTCGC
CAAAGCAAAAGAAGTTGGCGCAACCGAATGCGTGAATCCGCAGGATTATAAAAAACCGATTCAGGAAGTT
CTGACCGAAATGAGTAATGGCGGTGTTGACTTCAGCTTCGAAGTGATTGGCCGCCTGGATACCATGGTG
ACCGCACTGAGCTGCTGTCAGGAAGCCTATGGTGTGAGTGTGATTGTGGGTGTGCCGCCGGATAGTCA
GAATCTGAGCATGAATCCGATGCTGCTGCTGAGCGGTCGTACCTGGAAAGGTGCCATCTTCGGCGGCTT
CAAAAGTAAAGATAGTGTTCCGAAACTGGTGGCAGACTTCATGGCCAAAAAATTCGCACTGGATCCGCTG
ATTACCCATGTTCTGCCGTTCGAAAAAATTAATGAAGGCTTCGATCTGCTGCGTAGCGGCGAAAGCATTC
GCACCATTCTGACCTTC

Amino acid sequence of ADH1E

MSTAGKVIKCKAAVLWEEKKPFSIEEVEVAPPKAHEVRIKMVATGICRSDDHVVSGTLVTPLPVIAGHEAAGIV
ESIGEGVTTVRPGDKVIPLFTPQCGKCRVCKHPEGNFCLKNDLSMPRGTMQDGTSRFTCRGKPIHHFLGTS
TFSQYTVVDEISVAKIDAASPLEKVCLIGCGFSTGYGSAVKVAKVTQGSTCAVFGLGGVGLSVIMGCKAAGAA
RIIGVDINKDKFAKAKEVGATECVNPQDYKKPIQEVLTEMSNGGVDFSFEVIGRLDTMVTALSCCQEAYGVSVI
VGVPPDSQNLSMNPMLLLSGRTWKGAIFGGFKSKDSVPKLVADFMAKKFALDPLITHVLPFEKINEGFDLLRS
GESIRTILTF

DNA sequence of LB-ADH

ATGAGCAATCGTCTGGATGGTAAAGTTGCAATTATTACCGGTGGCACCCTGGGTATTGGTCTGGCAATT
GCAACCAAATTTGTTGAAGAAGGTGCCAAAGTGATGATTACCGGTCGTCATAGTGATGTTGGTGAAAA
AGCAGCAAAAAGCGTTGGTACACCGGATCAGATTCAGTTTTTTCAGCATGATAGCAGTGATGAAGATG
GTTGGACCAAACTGTTTGATGCAACCGAAAAAGCATTTGGTCCGGTTAGCACCCTGGTTAATAATGCA
GGTATTGCCGTTAATAAAAGCGTTGAAGAAACCACCACCGCAGAATGGCGTAAACTGCTGGCAGTTAA
TCTGGATGGTGTTTTTTTTGGCACCCGTCTGGGTATTCAGCGCATGAAAAATAAAGGTCTGGGTGCCA
GCATTATTAATATGAGCAGCATTGAAGGCTTTGTTGGTGATCCGAGCCTGGGTGCATATAATGCAAGCA
AAGGTGCAGTTCGCATTATGAGCAAAAGCGCAGCACTGGATTGTGCACTGAAAGATTATGATGTTCGT
GTGAATACCGTGCATCCGGGTTATATTAAAACACCGCTGGTTGATGATCTGCCTGGTGCCGAAGAAGC
AATGAGCCAGCGTACCAAAACCCCGATGGGTCATATTGGTGAACCGAATGATATTGCCTATATTTGTGT
TTATCTGGCCAGCAATGAAAGCAAATTTGCAACAGGTAGCGAATTTGTTGTGGATGGTGGTTATACCGC
ACAG

Amino acid sequence of LB-ADH

MSNRLDGKVAIITGGTLGIGLAIATKFVEEGAKVMITGRHSDVGEKAAKSVGTPDQIQFFQHDSSDEDGWT
KLFDATEKAFGPVSTLVNNAGIAVNKSVEETTTAEWRKLLAVNLDGVFFGTRLGIQRMKNKGLGASIINMSS
IEGFVGDPSLGAYNASKGAVRIMSKSAALDCALKDYDVRVNTVHPGYIKTPLVDDLPGAEEAMSQRTKTP
MGHIGEPNDIAYICVYLASNESKFATGSEFVVDGGYTAQ

DNA sequence of LK-ADH

ATGACTGACCGTTTGAAAGGTAAGGTAGCAATTGTAACTGGCGGTACCTTGGGAATTGGCTTGGCAATCG
CTGATAAGTTTGTTGAAGAAGGCGCAAAGGTTGTTATTACCGGCCGTCACGCTGATGTAGGTGAAAAAGC

TGCCAAATCAATCGGCGGCACAGACGTTATCCGTTTTGTCCAACACGATGCTTCTGATGAAGCCGGCTG
GACTAAGTTGTTTGATACGACTGAAGAAGCATTTGGCCCAGTTACCACGGTTGTCAACAATGCCGGAATT
GCGGTCAGCAAGAGTGTTGAAGATACCACAACTGAAGAATGGCGCAAGCTGCTCTCAGTTAACTTGGAT
GGTGTCTTCTTCGGTACCCGTCTTGGAATCCAACGTATGAAGAATAAAGGACTCGGAGCATCAATCATCA
ATATGTCATCTATCGAAGGTTTTGTTGGTGATCCAACTCTGGGTGCATACAACGCTTCAAAAGGTGCTGTC
AGAATTATGTCTAAATCAGCTGCCTTGGATTGCGCTTTGAAGGACTACGATGTTCGGGTTAACACTGTTCA
TCCAGGTTATATCAAGACACCATTGGTTGACGATCTTGAAGGGGCAGAAGAAATGATGTCACAGCGGACC
AAGACACCAATGGGTCATATCGGTGAACCTAACGATATCGCTTGGATCTGTGTTTACCTGGCATCTGACG
AATCTAAATTTGCCACTGGTGCAGAATTCGTTGTCGACGGTGGCTACACTGCTCAATAG

Amino acid sequence of LK-ADH

MTDRLKGKVAIVTGGTLGIGLAIADKFVEEGAKVVITGRHADVGEKAAKSIGGTDVIRFVQHDASDEAGWTKL
FDTTEEAFGPVTTVVNNAGIAVSKSVEDTTTEEWRKLLSVNLDGVFFGTRLGIQRMKNKGLGASIINMSSIEG
FVGDPTLGAYNASKGAVRIMSKSAALDCALKDYDVRVNTVHPGYIKTPLVDDLEGAEEMMSQRTKTPMGHIG
EPNDIAWICVYLASDESKFATGAEFVVDGGYTAQ

DNA sequence of LS-ADH

ATGGCACAGTATGATGTTGCAGATCGTAGCGCAATTGTTACCGGTGGTGGTAGCGGTATTGGTCGTGCAG
TTGCACTGACCCTGGCTGCAAGCGGTGCAGCAGTTCTGGTTACCGATCTGAATGAAGAACATGCACAGG
CAGTTGTTGCAGAAATTGAAGCAGCAGGCGGAAAAGCCGCTGCACTGGCTGGTGATGTTACCGATCCG
GCATTTGGTGAAGCAAGCGTTGCAGGTGCAAATGCACTGGCTCCGCTGAAAATTGCAGTTAACAATGCC
GGTATTGGTGGTGAAGCAGCAACCGTTGGTGATTACAGCCTGGATAGCTGGCGTACCGTTATTGAAGTTA
ATCTGAATGCCGTGTTTTATGGTATGCAGCCGCAGCTGAAAGCAATGGCAGCAAATGGTGGTGGTGCCAT
TGTTAATATGGCAAGCATTCTGGGTAGCGTTGGTTTTGCAAATAGCAGCGCCTATGTGACCGCAAAACAT
GCACTGCTGGGTCTGACACAGAATGCAGCACTGGAATATGCAGCAGATAAAGTTCGTGTTGTTGCAGTT
GGTCCGGGTTTTATTCGTACACCGCTGGTTGAAGCAAATCTGAGCGCAGATGCACTGGCCTTTCTGGAA
GGTAAACATGCCCTGGGTCGTCTGGGTGAACCGGAAGAAGTTGCAAGCCTGGTTGCATTTCTGGCCTCT
GATGCAGCAAGCTTTATTACCGGTAGCTATCATCTGGTTGATGGTGGTTATACCGCACAG

Amino acid sequence of LS-ADH

MAQYDVADRSAIVTGGGSGIGRAVALTLAASGAAVLVTDLNEEHAQAVVAEIEAAGGKAAALAGDVTDPAFGE
ASVAGANALAPLKIAVNNAGIGGEAATVGDYSLDSWRTVIEVNLNAVFYGMQPQLKAMAANGGGAIVNMASIL
GSVGFANSSAYVTAKHALLGLTQNAALEYAADKVRVVAVGPGFIRTPLVEANLSADALAFLEGKHALGRLGEP
EEVASLVAFLASDAASFITGSYHLVDGGYTAQ

DNA sequence of RR-ADH

ATGAAAGCCCTCCAGTACACCGAGATTGGCAGCGAACCGGTGGTGGTGGATGTTCCGACGCCGGCACC
GGGTCCGGGCGAAATTCTGCTGAAAGTGACGGCGGCAGGCCTGTGCCATAGCGATATTTTTGTGATGGA
TATGCCGGCGGAACAGTATATTTATGGCCTGCCGCTGACCCTGGGCCATGAAGGCGTTGGCACGGTGGC
GGAACTGGGCGAAGGCGTGACGGGCTTTGAAACGGGCGATGCGGTGGCGGTTTATGGCCCGTGGGGC
TGCGGCGCGTGTCATGCGTGTGCGCGTGGCCGCGAAAATTATTGCACGCGTGCGGCCGAACTGGGCAT
TACTCCGCCGGGTCTGGGCAGCCCGGGCAGCATGGCGGAATACATGATTGTGGATAGCGCGCGTCATC
TGGTGCCGATTGGCGATCTGGACCCGGTTGCGGCGGTTCCGCTGACGGATGCGGGCCTGACGCCGTAT
CATGCGATTAGCCGCGTGCTGCCGCTGCTGGGTCCGGGTAGCACGGCCGTGGTTATTGGCGTTGGCGG
CCTGGGCCATGTGGGCATTCAGATTCTGCGCGCGGTTTCTGCGGCACGTGTGATTGCGGTGGATCTGG
ATGATGATCGCCTGGCCCTGGCCCGTGAAGTTGGCGCGGATGCGGCGGTTAAAAGCGGCGCAGGCGC
AGCGGATGCGATTCGTGAACTGACTGGCGGCGAAGGCGCCACGGCGGTGTTTGATTTTGTGGGCGCG
CAGAGCACGATTGATACGGCGCAGCAGGTGGTGGCGATTGATGGCCATATTAGCGTGGTGGGCATTCAT
GCAGGCGCGCATGCGAAAGTGGGCTTTTTTATGATTCCGTTTGGCGCGAGCGTGGTGACGCCGTATTGG
GGCACGCGCAGCGAACTGATGGATGTGGTGGATCTGGCCCGTGCGGGCCGTCTGGATATTCATACGGA
AACGTTTACCCTGGATGAAGGCCCGACGGCGTATCGCCGTCTGCGCGAAGGCAGCATTCGTGGCCGTG
GCGTGGTGGTGCCGGGCTAA

Amino acid sequence of RR-ADH

MKALQYTEIGSEPVVVDVPTPAPGPGEILLKVTAAGLCHSDIFVMDMPAEQYIYGLPLTLGHEGVGTVAELGE
GVTGFETGDAVAVYGPWGCGACHACARGRENYCTRAAELGITPPGLGSPGSMAEYMIVDSARHLVPIGDLD
PVAAVPLTDAGLTPYHAISRVLPLLGPGSTAVVIGVGGLGHVGIQILRAVSAARVIAVDLDDDRLALAREVGADA
AVKSGAGAADAIRELTGGEGATAVFDFVGAQSTIDTAQQVVAIDGHISVVGIHAGAHAKVGFFMIPFGASVVTP
YWGTRSELMDVVDLARAGRLDIHTETFTLDEGPTAYRRLREGSIRGRGVVVPG

DNA sequence of SmNOX

ATGAGTAAGATCGTGATTGTTGGCGCCAATCATGCCGGTACCGCAGCAATTAATACCGTTCTGGATAATTA
TGGTAGTGAAAATGAAGTGGTGGTGTTTGATCAGAATAGCAATATTAGCTTTCTGGGTTGTGGCATGGCA
CTGTGGATTGGCAAACAGATTAGTGGTCCGCAGGGCCTGTTTTATGCAGATAAAGAAAGTCTGGAAGCC
AAAGGCGCCAAAATCTATATGGAAAGCCCGGTTACCGCAATTGATTATGATGCAAAACGTGTTACCGCCCT
GGTTAATGGCCAGGAACATGTGGAAAGCTATGAAAAACTGATTCTGGCCACCGGTAGTACCCCGATTCTG
CCGCCGATTAAGGGTGCAGCCATTAAGGAAGGTAGTCGCGATTTTGAAGCCACCCTGAAAAATCTGCAG
TTTGTTAAACTGTATCAGAATGCAGAAGATGTTATTAATAAGCTGCAGGATAAAACCCAGAATCTGAATCGT
ATTGCCGTTGTGGGCGCCGGCTATATTGGTGTGGAACTGGCAGAAGCCTTTAAACGTCTGGGTAAAGAA
GTGATTCTGATTGATCGCCATGATACCTGTCTGGCCGGTTATTATGATCAGGATCTGAGTGAAATGATGCG
CCAGAATCTGGAAGATCATGGCATTGAACTGGCATTTGGCGAAACCGTTAAAGCCATTGAAGGCGATGG
CAAAGTGGAACGTATTGTGACCGATAAAGCCAGTCATGATGTTGATATGGTTATTCTGGCAGTTGGCTTTC
GCCCGAATACCGCCCTGGGTAATGCAAAACTGAAAACCTTTCGTAATGGCGCATTTCTGGTTGATAAAAA
ACAGGAAACCAGTATTCCGGATGTGTATGCAATTGGCGATTGTGCAACCGTGTATGATAATGCCATTAATG
ATACCAATTACATCGCCCTGGCAAGCAATGCACTGCGCAGTGGTATTGTTGCAGGCCATAATGCCGCAGG
CCATAAACTGGAAAGCCTGGGTGTTCAGGGTAGCAATGGCATTAGCATTTTTGGTCTGAATATGGTGAGC
ACCGGTCTGACCCAGGAAAAAGCAAAACGTTTTGGCTATAATCCGGAAGTGACCGCCTTTACCGATTTTC
AGAAAGCCAGTTTTATTGAACATGATAATTACCCGGTTACCCTGAAAATTGTGTATGATAAAGATAGCCGTC
TGGTGCTGGGCGCACAGATGGCAAGCAAAGAAGATATGAGTATGGGTATTCACATGTTTAGCCTGGCAAT
TCAGGAAAAAGTTACCATTGAACGTCTGGCACTGCTGGATTATTTCTTTCTGCCGCATTTTAATCAGCCGT
ATAATTATATGATCAAGGCCGCACTGAAAGCAAAA

Amino acid sequence of SmNOX

MSKIVIVGANHAGTAAINTVLDNYGSENEVVVFDQNSNISFLGCGMALWIGKQISGPQGLFYADKESLEAKGA
KIYMESPVTAIDYDAKRVTALVNGQEHVESYEKLILATGSTPILPPIKGAAIKEGSRDFEATLKNLQFVKLYQNAE
DVINKLQDKTQNLNRIAVVGAGYIGVELAEAFKRLGKEVILIDRHDTCLAGYYDQDLSEMMRQNLEDHGIELAF
GETVKAIEGDGKVERIVTDKASHDVDMVILAVGFRPNTALGNAKLKTFRNGAFLVDKKQETSIPDVYAIGDCAT
VYDNAINDTNYIALASNALRSGIVAGHNAAGHKLESLGVQGSNGISIFGLNMVSTGLTQEKAKRFGYNPEVTA
FTDFQKASFIEHDNYPVTLKIVYDKDSRLVLGAQMASKEDMSMGIHMFSLAIQEKVTIERLALLDYFFLPHFNQ
PYNYMIKAALKAK
